# Transcriptional, epigenetic and retroviral signatures identify regulatory regions involved in hematopoietic lineage commitment

**DOI:** 10.1038/srep24724

**Published:** 2016-04-20

**Authors:** Oriana Romano, Clelia Peano, Guidantonio Malagoli Tagliazucchi, Luca Petiti, Valentina Poletti, Fabienne Cocchiarella, Ermanno Rizzi, Marco Severgnini, Alessia Cavazza, Claudia Rossi, Pasqualepaolo Pagliaro, Alessandro Ambrosi, Giuliana Ferrari, Silvio Bicciato, Gianluca De Bellis, Fulvio Mavilio, Annarita Miccio

**Affiliations:** 1 Department of Life Sciences, University of Modena and Reggio Emilia, Modena, Italy; 2 Center for Genomic Research, University of Modena and Reggio Emilia, Modena, Italy; 3 INSERM UMR 1163, Laboratory of chromatin and gene regulation during development, Paris, France; 4 Institute of Biomedical Technologies, CNR, Milan, Italy; 5 Genethon, Evry, France; 6 Telethon Foundation, Milan, Italy; 7 Dana Farber Cancer Institute, Harvard Medical School, Boston, US; 8 San Raffaele-Telethon Institute for Gene Therapy (TIGET), San Raffaele Scientific Institute, Milan, Italy; 9 Az. Osp. Policlinico Universitario di Bologna, Policlinico S. Orsola-Malpighi, Unità Operativa di Immunoematologia e Trasfusionale, Bologna, Italy; 10 Vita Salute San Raffaele University, Milan, Italy; 11 Paris Descartes, Sorbonne Paris Cité University, Imagine Institute, Paris, France

## Abstract

Genome-wide approaches allow investigating the molecular circuitry wiring the genetic and epigenetic programs of human somatic stem cells. Hematopoietic stem/progenitor cells (HSPC) give rise to the different blood cell types; however, the molecular basis of human hematopoietic lineage commitment is poorly characterized. Here, we define the transcriptional and epigenetic profile of human HSPC and early myeloid and erythroid progenitors by a combination of Cap Analysis of Gene Expression (CAGE), ChIP-seq and Moloney leukemia virus (MLV) integration site mapping. Most promoters and transcripts were shared by HSPC and committed progenitors, while enhancers and super-enhancers consistently changed upon differentiation, indicating that lineage commitment is essentially regulated by enhancer elements. A significant fraction of CAGE promoters differentially expressed upon commitment were novel, harbored a chromatin enhancer signature, and may identify promoters and transcribed enhancers driving cell commitment. MLV-targeted genomic regions co-mapped with cell-specific active enhancers and super-enhancers. Expression analyses, together with an enhancer functional assay, indicate that MLV integration can be used to identify *bona fide* developmentally regulated enhancers. Overall, this study provides an overview of transcriptional and epigenetic changes associated to HSPC lineage commitment, and a novel signature for regulatory elements involved in cell identity.

The gene expression program of stem/progenitor cells and their progeny is temporally controlled by the coordinated action of transcription factors (TFs) that bind to DNA regulatory elements, including promoters and enhancers. TFs regulate gene expression and at the same time shape the epigenetic state of chromatin by recruiting DNA methylation and histone modification complexes playing essential roles during commitment^1^,^2^. Promoters and enhancers are associated to specific chromatin signatures generated by histone modifications^3^. In particular, the combination of histone H3 methylation and acetylation profiles allows the definition of strong and weak promoters and enhancers^3^,^4^,^5^,^6^,^7^. Super-enhancers were recently defined as clusters of acetylated enhancers, which are densely occupied by TFs and control the expression of genes defining cell identity^8^,^9^.

Genome-wide approaches allow to analyze the functional relationships between chromatin dynamics, gene expression patterns and cell phenotype with unprecedented levels of detail and integration. Most of the available knowledge on the molecular mechanisms driving stem cell development, commitment and differentiation comes from the study of murine models^10^,^11^, embryonic stem cells^12^,^13^,^14^,^15^,^16^,^17^, or terminally differentiated cells^5^,^18^,^19^,^20^,^21^,^22^,^23^,^24^,^25^,^26^, while much less is known about primary somatic stem/progenitor cells, particularly in a human context^18^,^19^,^27^,^28^,^29^,^30^,^31^. The differential usage of regulatory regions by hematopoietic stem cells, multipotent and committed progenitors was recently defined in the murine system^10^,^11^. The transcriptome and epigenome of differentiated hematopoietic cells were extensively analyzed also in the human system^32^,^33^,^34^. However, information on promoter, enhancer and super-enhancer usage in the early phases of human lineage differentiation is still lacking, and necessary to unravel the mechanisms driving early decisions on commitment and lineage restriction by a multipotent progenitor.

This study describes the transcriptional and epigenetic profile of human HSPC and their early committed erythroid and myeloid progeny, as determined by CAGE, ChIP-seq for histone modifications, and retroviral scanning, a novel tool to map active regulatory sequences based on the integration properties of the Moloney murine leukemia virus (MLV). MLV integrates almost exclusively in active promoters and enhancers in mammalian genomes^35^,^36^,^37^, as a consequence of the binding of the viral integrase to the bromodomain and extraterminal protein BRD4, which tethers the pre-integration complex to chromatin regions rich in acetylated histones^38^,^39^,^40^,^41^. High-definition maps of MLV integration sites (“integromes”) therefore provide an additional, functional tool to identify active regulatory regions in cell development and differentiation. We integrated data coming from transcriptome, epigenome and integrome analysis in coherent maps that describe the differential genetic and epigenetic programs of HSPC and committed progenitors/precursors, and define known as well as novel promoters, enhancers and super-enhancers associated with erythroid and myeloid commitment. Finally, we show that MLV integration clusters target *bona fide* cell-specific enhancers and genes defining cell identity.

## Results

### Purification and characterization of multipotent and lineage-restricted hematopoietic progenitors

**Figure 1:**
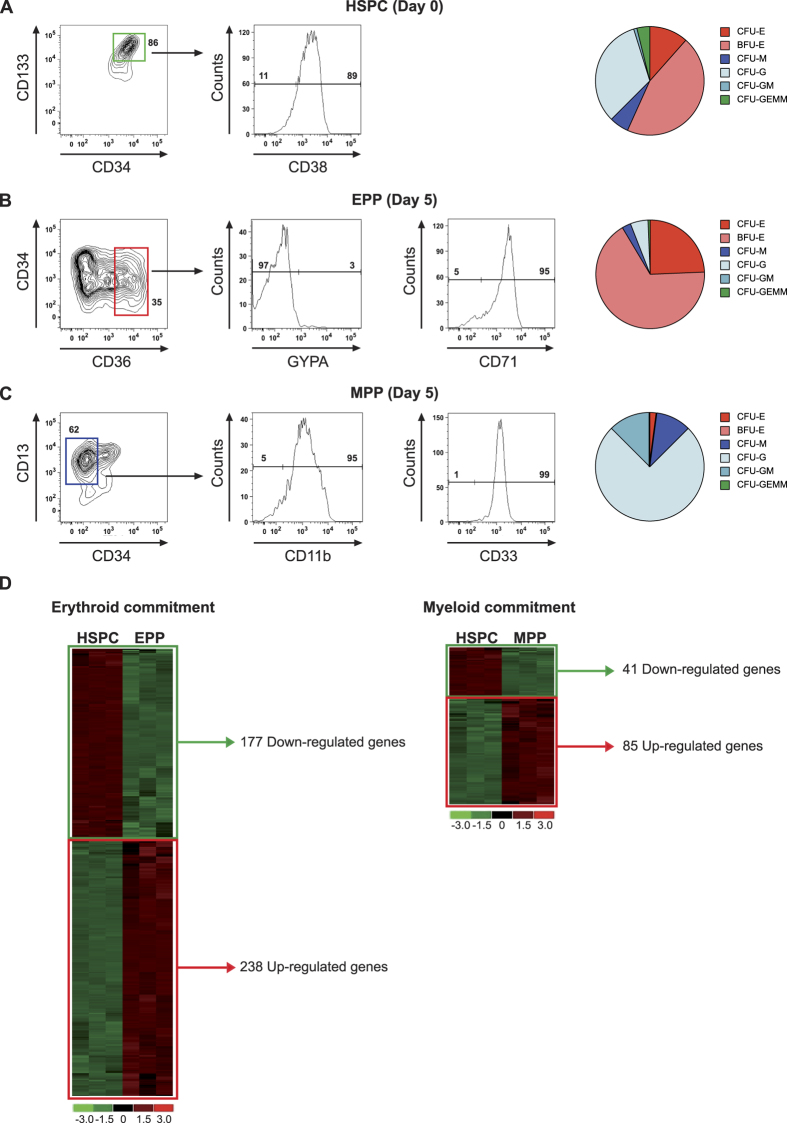
Purification and characterization of multipotent and lineage-restricted hematopoietic progenitors. (**A**–**C**) FACS analysis and CFC assay of HSPC, EPP and MPP. Under conditions supporting erythroid differentiation, EPP differentiated into late erythrocytes (CD71^+^/GYPA^+^), while MPP remained in an undifferentiated myeloid state (CD11b^low^/CD14^low^/CD71^low^). In the presence of G-CSF, MPP differentiated into mature myeloid cells (CD11b^+^/CD14^+^) while EPP expressed low levels of late myeloid markers (CD11b^low^/CD14^low^) (**D**) HSPC, EPP and MPP gene expression profiles. Supervised analysis was performed using a fold-change threshold equal to 2 and a *P*-value threshold equal to 0.05, to obtain a list of differentially-expressed genes.

We prospectively enriched human HSPC as CD34^+^/CD133^+^ population by FACS (Fig. 1A). HSPC showed high levels of CD38, indicating that the majority of the cells were early hematopoietic progenitors^42^ (Fig. 1A). Committed erythroid and myeloid progenitors/precursors (EPP and MPP) were isolated as CD34^low^/CD36^high^ and CD34^−^/CD13^+^ populations (Fig. 1B,C). Over 95% of EPP were CD71^+^ and expressed low levels of glycophorin A (GYPA), indicating that they are mainly composed by erythroid progenitors^32^ (Fig. 1B). MPP expressed the myeloid differentiation markers CD33 and CD11b (Fig. 1C)^43^ and low levels of the late differentiation marker CD14 (not shown). In a clonal progenitor assay, HSPC gave rise to mixed colonies (CFU-GEMM), and both myeloid (CFU-GM, CFU-G, CFU-M) and erythroid (BFU-E and CFU-E) colonies, thus confirming their multilineage potential (Fig. 1A). In contrast, EPP and MPP populations generated >90% erythroid and myeloid colonies, respectively (Fig. 1B,C), confirming their lineage-restricted potential. EPP and MPP populations were grown in liquid culture under conditions supporting either erythroid (+EPO) or myeloid (+G-CSF) terminal differentiation. In the presence of EPO, EPP were able to differentiate into late erythrocytes, while MPP remained in an undifferentiated myeloid state (Supplementary Fig. 1A). Conversely, in the presence of G-CSF, MPP differentiate into granulocytes and monocytes while EPP grew poorly and acquired late myeloid markers (Supplementary Fig. 1B).

HSPC, EPP and MPP were further characterized by gene expression profiling on standard Affymetrix microarrays. Principal component analysis (PCA) of gene expression profiles showed that biological replicates of each cell population were reproducible, and that HSPC, EPP and MPP exhibit distinct gene expression profiles (Supplementary Fig. 1C). Supervised analysis using dChip (fold-change > 2, p-value < 0.05) identified 415 and 126 differentially expressed (DE) genes upon erythroid and myeloid commitment of HSPC (Fig. 1D). Genes down-regulated in both conditions belong to TGFβ/BMP signaling pathways implicated in HSC self-renewal (*NOG*, *CHRDL1*, *TGFB1I1*), or to the tumor necrosis factor superfamily, involved in T- and B-cell functions (*TNFSF13B*, *TNFSF4*, *LTB*), while genes involved in leukocyte biology (*MPO*, *CTSG*, *GZMA*) were down-regulated upon erythroid commitment. Genes up-regulated in EPP are involved in erythrocyte differentiation and homeostasis and include the master regulators *GATA1* and *KLF1*. Instead, genes up-regulated in MPP are mainly involved in immune defense function of neutrophils and macrophages (e.g., *ELANE*, *AZU1*, *CTSG*) (Supplementary Table 1).

### Defining transcription initiation in HSPC and lineage-restricted progenitors

**Figure 2:**
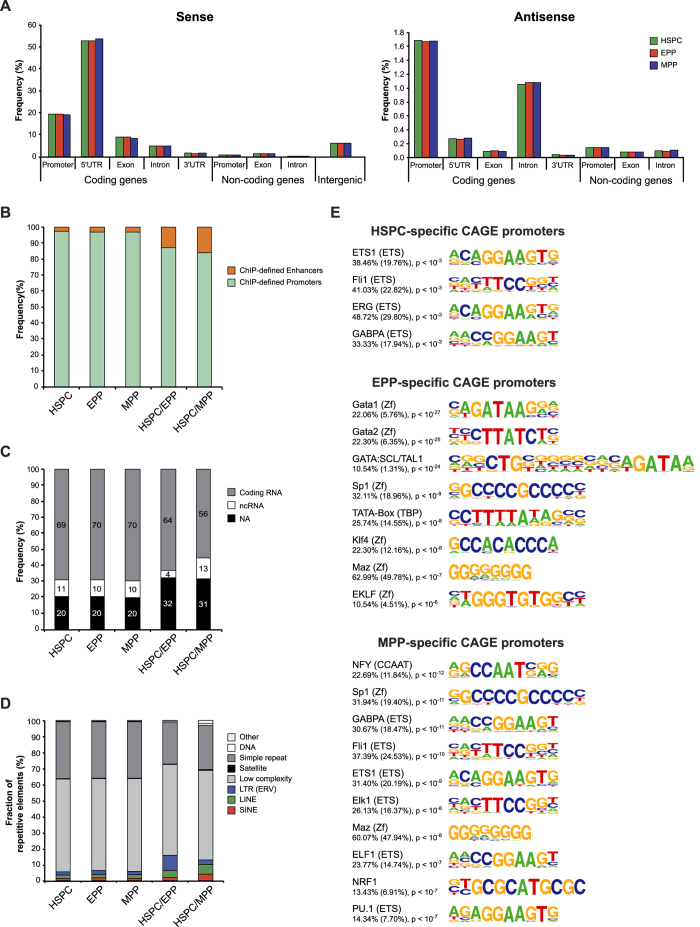
Analysis of CAGE promoters. (**A**) Genomic distribution of CAGE TSSs in HSPC, EPP and MPP. TSSs were mapped to regions annotated as promoters (500 bp-long regions upstream annotated TSSs), 5′ UTR, exon, intron and 3′ UTR of coding and noncoding genes (in sense or antisense orientation) or as intergenic regions. For each category, frequency is indicated. (**B**) Distribution of total and differentially used CAGE promoters overlapping with epigenetically defined promoters and enhancers. (**C**) Annotation of total and differentially used CAGE promoters. The graphs show the proportions of total and differentially used CAGE promoters associated to coding RNA and ncRNA (miRNA, rRNA, snoRNA and snRNAand lincRNA). (**D**) Distribution of total and differentially used CAGE promoters amongst the different classes of repetitive elements, defined by RepeatMasker. Total CAGE promoters: HSPC, EPP and MPP. Differentially used CAGE promoters: HSPC/EPP and HSPC/MPP. (**E**) Top enriched TF motifs within CAGE promoters (−300 to +100 bp from TSSs). Transcription factor motif finding in cell-specific promoters was performed using HOMER software. The frequency of target (background) sequences enriched in TF motifs and p-values are indicated.

To define the promoter usage in HSPC and their committed progeny, we used Cap Analysis of Gene Expression (CAGE), a technique that identifies active transcription start sites (TSSs) at single base-pair resolution and measures the expression level of each transcript^44^. We clustered CAGE tags into 2 levels: Level-1 promoters (“TSSs”) were created by summing the weighted number of CAGE tags that have an identical 5′ start site, and were then clustered in Level-2 promoters (“CAGE promoters”) if they were within 20 bp of each other and had similar expression levels. We mapped by CAGE ~0.6 × 10^6^ TSSs in each cell population, typically scattered over short genomic regions due to the inherent variability of transcription initiation^45^. As an example, 3 TSSs were mainly used to drive transcription of the human beta globin gene (*HBB*) in EPP, which started at low frequency at 13 nucleotide positions in a 58-bp region encompassing the 5′ UTR of *HBB* (Supplementary Fig. 2). We mapped most of the TSSs (>70%) to regions annotated as promoters and 5′ UTR of known transcripts (Fig. 2A). Interestingly, 23% of TSSs were mapped to intergenic regions, exons, introns, and 3′ UTR, suggesting the presence of alternative or novel, yet unannotated promoters (Fig. 2A). Notably, TSSs mapping to exons, introns and 3′ UTRs of coding genes had lower expression levels compared to those mapping to promoters and 5′ UTRs (Supplementary Fig. 3A). About 3.5% of TSSs mapped to the antisense strand of known genes, mostly in promoters and introns (Fig. 2A).

We defined >13,000 CAGE promoters of similar average length (160 ± 101 bp) in each cell type as clusters of nearby TSSs (13,852 in HSPC, 13,609 in MPP and 14,041 in EPP), >96% of which overlapping with epigenetically defined promoters (Fig. 2B). We assigned CAGE promoters to the closest transcript using publicly available datasets. The majority (~80%) of CAGE promoters were annotated to known genes and particularly to protein-coding transcripts (Fig. 2C). A correlation of the microarray expression values with the tpm counts of all CAGE transcripts associated to known genes showed a statistically significant concordance between the two data sets (Pearson’s r ~ 0.54) (Supplementary Fig. 3B). Transcription of ~10% of known genes was driven >2 alternative promoters (Supplementary Table 2). Interestingly, >2,600 promoters (~20%) were not associated with known genes (Fig. 2C), and may drive transcription of yet unknown transcripts. About 24% of CAGE promoters overlapped with repetitive elements, ~6% of which were transposable elements (LINE, SINE and LTR) (Fig. 2D).

### A minority of active promoters is differentially expressed during lineage commitment of HSPC

To determine the differential promoter usage during hematopoietic lineage commitment, we identified 714 differentially used (DU) promoters between HSPC and EPP (306 down-regulated and 408 up-regulated) and 1,127 between HSPC and MPP (576 down-regulated and 551 up-regulated). We observed a high degree of correlation between DU CAGE promoters and DE genes: >40% DE genes were associated with DU CAGE annotated promoters. Moreover, we validated differential RNA expression by qRT-PCR in a sample of 18 DU promoters (Supplementary Fig. 4). Only 78 promoters were expressed exclusively in HSPC and down-regulated in both EPP and MPP (“HSPC-specific”), while the remaining down-regulated promoters remained expressed in one of the two lineages. On the contrary, the vast majority of up-regulated promoters were lineage-specific: 399 out of 408 were upregulated in EPP only (“EPP-specific”), and 522 out of 551 in MPP only (“MPP-specific”). Notably, 14% of DU CAGE promoters overlapped with epigenetically defined enhancers, a significantly higher proportion compared to total CAGE promoters (3%, *P* < 0.0001) (Fig. 2B), and 30% were not associated to known genes compared to 20% for total promoters (*P* < 0.01) (Fig. 2C). DU promoters mapped to transposable elements more frequently than total CAGE promoters (14% *vs*. 6%, *P* < 0.0001) (Fig. 2D), as well as to intergenic and intronic regions (Supplementary Fig. 5).

We observed lineage-specific alternative promoter usage only for 6 genes. As an example, the *LMO2* gene, coding for a developmentally regulated TF, is transcribed from 3 different promoters, of which Promoter 3 was active only in HSPC, Promoter 1 mainly in HSPC and MPP, and Promoter 2 predominantly in EPP (Supplementary Fig. 6).

A significant number of genes associated with CAGE promoters were functionally linked in cell-specific networks, e.g., DNMT3A and CD34 pathways for HSPC-specific and down-regulated promoters, and the GATA1 and CSF3R pathways for EPP- and MPP-specific promoters, respectively (Supplementary Fig. 7A). Genes transcribed by HSPC-specific promoters were enriched in functional categories related to multicellular organismal development and immune response (Supplementary Fig. 7B). Genes associated to EPP- and MPP-specific promoters were mainly involved in erythroid development and leukocyte biology, respectively (Supplementary Fig. 7B). Conversely, promoters down-regulated in EPP were associated with genes involved in leukocyte biology, while those down-regulated in MPP were associated to translation and macromolecular complex organization (Supplementary Fig. 7B).

We then looked at the set of DU promoters driving the expression of TFs, co-factors and chromatin modifiers (Supplementary Fig. 4 and Supplementary Table 3). A few factors were highly expressed only in HSPC, such as the HSC regulators MYCN and DNMT3A, HOXA7, an essential TF in hematopoietic progenitors, SLA2, implicated in lymphocyte biology and NAP1L3 (nucleosome assembly protein 1-like 3), the function of which in hematopoiesis is yet unknown. EPP- and MPP-specific promoters drove the expression of known erythroid and myeloid transcriptional regulators, such as TAL1, GATA1 and KLF1 in EPP, and NFIA, KLF4 and STAT6 in MPP. However, more than 50% of EPP- and MPP-specific TFs and co-factors were not previously associated to erythropoiesis or myelopoiesis (Supplementary Table 3).

To better understand the regulatory circuitry operating on lineage-specific CAGE promoters, we analyzed putative TF binding sites (TFBS) within the proximal regions of cell-specific promoters. HSPC-specific promoters were fairly enriched for binding motifs of the ETS family of TFs, which regulate development and maintenance of HSCs and their differentiation along multiple lineages (Fig. 2E). EPP- and MPP-specific promoters were enriched for motifs of ubiquitous promoter-associated TFs, like SP1 and TBP, and lineage-specific TFs, such as GATA1, TAL1 and KLF1 (EKLF) for EPP, and GABPA, FLI1, and PU.1 for MPP (Fig. 2E).

**Figure 3:**
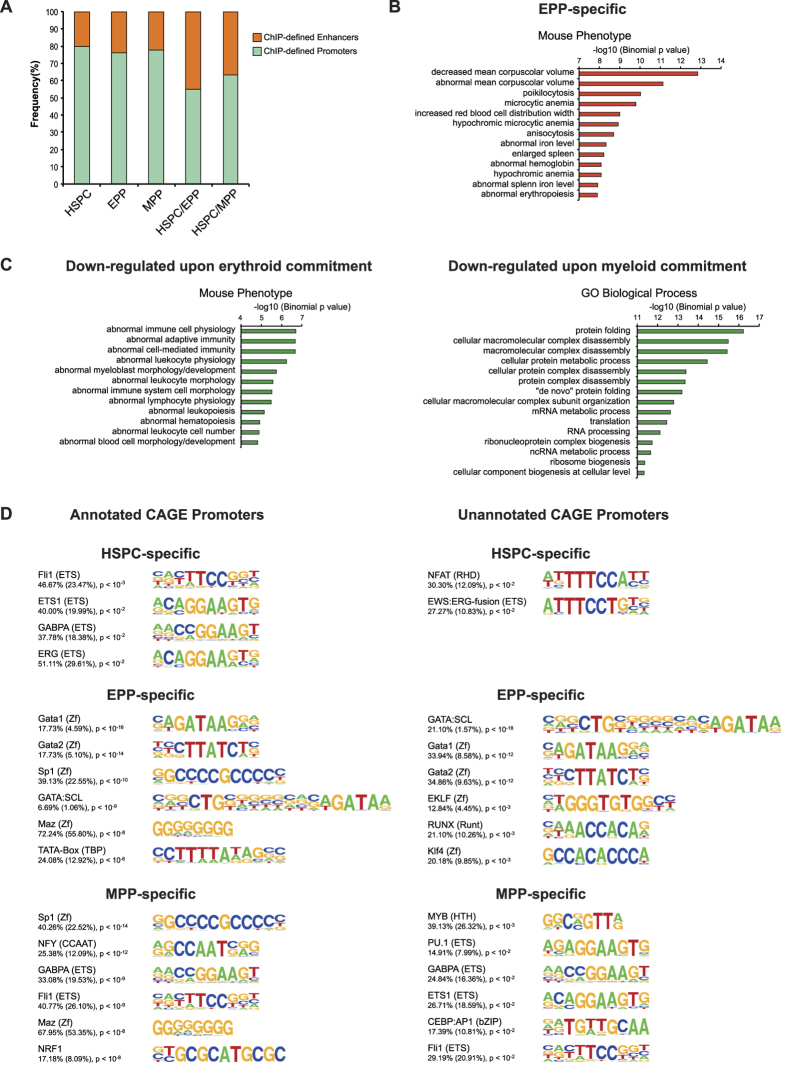
Analysis of novel CAGE promoters. (**A**) Distribution of total and differentially used unannotated CAGE promoters overlapping with epigenetically defined promoters and enhancers. (**B**,**C**) Gene ontology analysis of unannotated CAGE promoters. CAGE promoters (EPP- specific and down-regulated upon HSPC commitment), which were not assigned to any known gene or transcript, were analyzed using GREAT. (**D**) Top enriched TF motifs within annotated and unannotated CAGE promoters. Cell-specific annotated and unannotated CAGE promoters were enriched for similar cell-specific and ubiquitous transcription factor motifs. TF motif analysis was performed using HOMER. The frequency of target (background) sequences enriched in TF motifs and p-values are indicated.

Next, we analyzed CAGE promoters not assigned to known genes. Around 22% of these unannotated promoters harbored an epigenetic enhancer signature, a frequency that increased up to 45% for DU promoters (Fig. 3A), suggesting that they may represent regulatory regions associated with enhancer-derived RNA acting in cis on adjacent target genes. Regions surrounding EPP-specific unannotated promoters were associated with erythroid phenotypes (Fig. 3B), while those surrounding promoters down-regulated upon erythroid and myeloid commitment were associated with leukocyte phenotypes and RNA processing/macromolecular complex assembly, respectively (Fig. 3C). Finally, both annotated and unannotated promoters were enriched for the same ubiquitous and cell-specific TF motifs (Fig. 3D), indicating that novel promoters are in fact regulated by the same TFs binding to annotated promoters.

### Genome-wide histone modification profiling reveals that the majority of enhancers are differentially used in HSPC and lineage-restricted progenitors

To obtain a genome-wide description of chromatin changes occurring upon HSPC commitment, we used H3K4me3 and H3K4me1 to define putative promoters and enhancers, and H3K27ac to distinguish strong (H3K27ac^+^) from weak/inactive (H3K27ac^−^) regulatory elements. We identified >12,000 promoter regions in HSPC, EPP and MPP, with a similar average size, 60% of which carried the H3K27ac mark, and >44% were associated with CAGE TSSs (Supplementary Table 4). Most of H3K27ac^+^ promoters (>72%) were actively transcribed, and of these, 12% were not annotated, suggesting the presence of yet uncharacterized genes. In parallel, we defined >49,000 putative enhancers in both multipotent and committed progenitors (Supplementary Table 5). Roughly a quarter of enhancers were enriched in H3K27ac and only a minor fraction (<1%) was associated with CAGE unannotated promoters (Supplementary Table 5). These transcribed regions were more likely to be marked by H3K27ac and could represent eRNA-associated enhancers.

**Figure 4:**
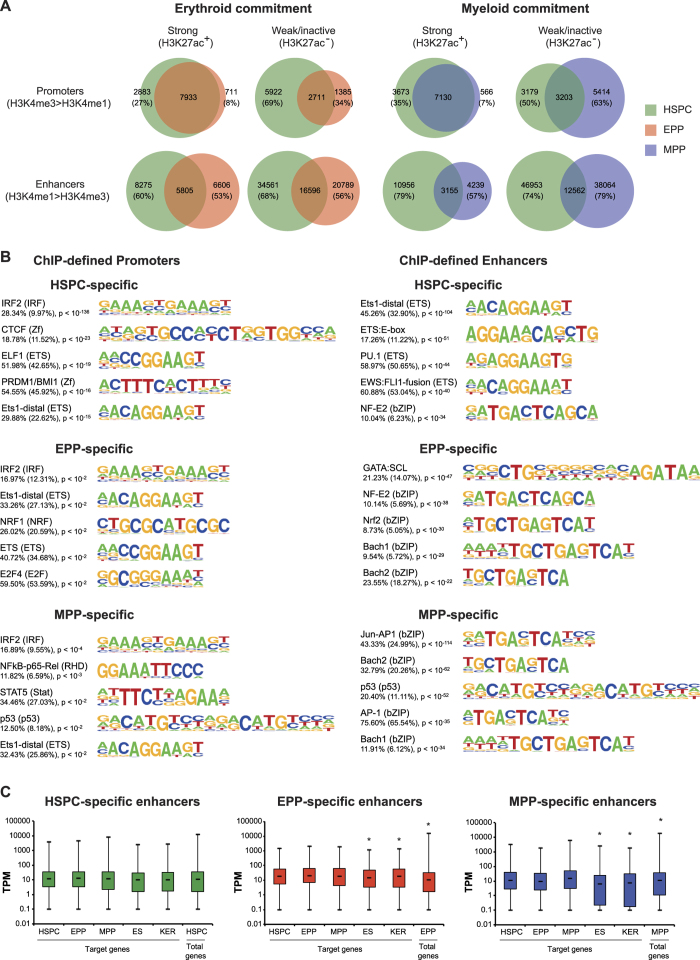
Analysis of epigenetically defined regulatory regions. (**A**) Dynamics of promoter and enhancer chromatin signatures upon HSPC commitment. Venn diagrams show the overlap of strong (H3K27ac^+^) and weak/inactive (H3K27ac^−^) promoters (H3K4me3 > H3K4me1) and enhancers (H3K4me1 > H3K4me3) identified in HSPC, EPP and MPP. The fraction of non-overlapping HSPC, EPP and MPP regulatory regions is indicated. Overall, we identified 7,107, 1,026 and 2,675 cell-specific promoters and 35,318, 19,465 and 43,120 cell-specific enhancers in HSPC, EPP and MPP, respectively. (**B**) Top enriched TF motifs in epigenetically defined regulatory regions. Putative TFBS in cell-specific promoters and enhancers were identified using HOMER. The frequency of target (background) sequences enriched in TF motifs and p-values are indicated. (**C**) Expression levels of CAGE promoters surrounding HSPC-, EPP- and MPP-specific enhancers (±5 kb interval) in HSPC, EPP, MPP, embryonic stem cells (ES) and keratinocytes (KER). As control, expression levels of total HSPC, EPP and MPP CAGE promoters were analyzed. A t-test was used to determine significant differences in the expression values associated to CAGE promoters in the different cell types (*P < 0.05). The median expression levels of genes close to weak/inactive enhancers was similar among the different cell populations (data not shown). Comparable results were obtained by analyzing the expression levels of CAGE promoters in a 100-Kb window (data not shown).

Next, we evaluated the dynamics of ChIP-defined regulatory elements upon HSPC commitment. The vast majority of strong EPP and MPP promoter regions (92% and 93%, respectively), and a low proportion of the weak/inactive ones, were shared with HSPC (Fig. 4A). On the contrary, a much lower proportion of both strong and weak enhancers were shared upon lineage commitment while the majority was cell-specific (Fig. 4A), suggesting that enhancers play a major role in HSPC commitment. Overall, we identified 7,107, 1,026 and 2,675 cell-specific promoters and 35,318, 19,465 and 43,120 cell-specific enhancers in HSPC, EPP and MPP, respectively. ChIP-defined cell-specific regulatory regions were modestly enriched in binding motifs of general and hematopoietic TFs, the majority of which were shared by the three populations (Fig. 4B).

To analyze the influence of H3K27ac^+^ cell-specific enhancers on nearby genes, we analyzed the expression level of the closest CAGE promoters. We observed a mild increase in the expression level of promoters close to active enhancers in all three cell population compared to unrelated cell populations, and at least in EPP and MPP, a significant increase compared to total CAGE promoters used by these populations (Fig. 4C). Functional annotation of genes targeted by active enhancers showed a low enrichment for cell-specific gene ontology categories (Supplementary Fig. 8).

### Super-enhancers are specific in HSPC and their committed progeny

**Figure 5:**
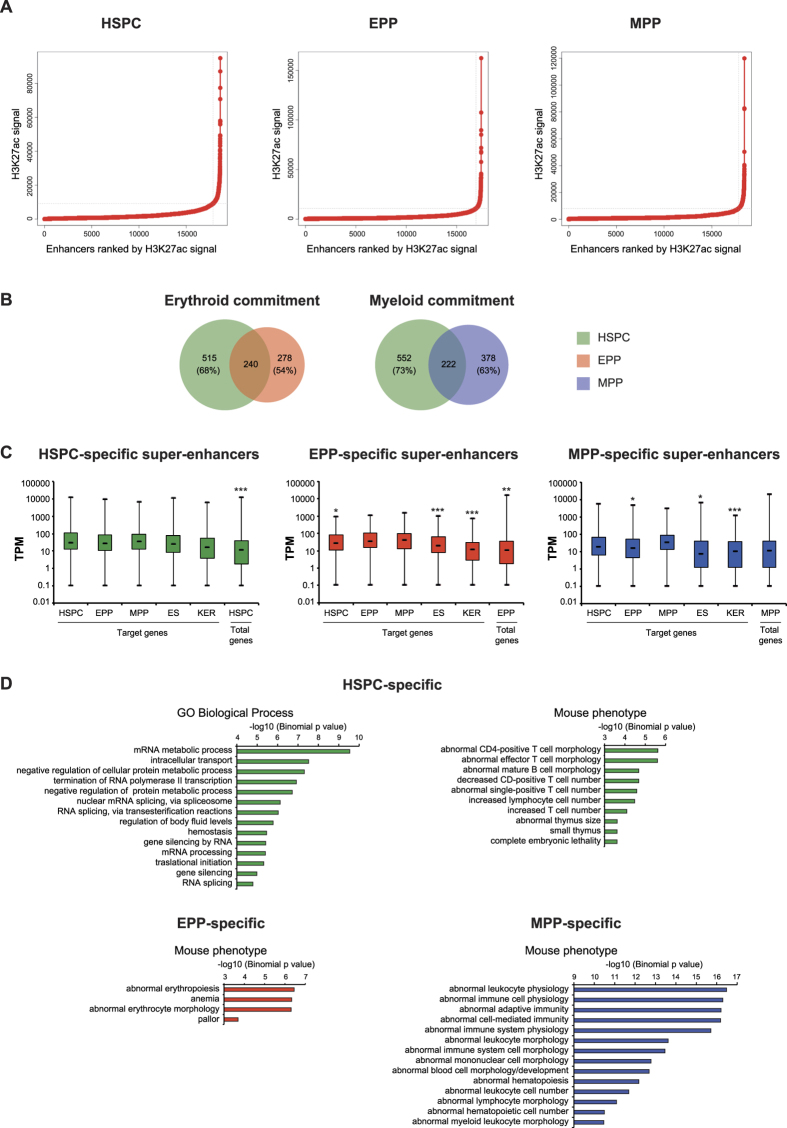
Analysis of super-enhancers. (**A**) The plots represents the distribution of H3K27ac ChIP-seq signal (in units of reads per million, on y-axis) across all the H3K27ac-containing enhancers (x-axis). Enhancers were ranked by increasing H3K27ac ChIP-seq signal. H3K27ac is not evenly distributed across the enhancer regions, with a subset of enhancers containing exceptionally high amounts of H3K27ac (SEs), as defined by surpassing the inflection point. (**B**) Differential SE usage upon erythroid and myeloid commitment. Venn diagrams showed the fraction of non-overlapping HSPC, EPP and MPP SEs. Overall, we identified 415 HSPC-specific, 245 EPP-specific and 337 MPP-specific SEs. (**C**) Expression levels of CAGE promoters driving the expression of genes targeted by cell-specific SE were analyzed in HSPC, EPP, MPP, embryonic stem cells (ES) and keratinocytes (KER). As control, we analyzed the expression levels of total HSPC, EPP and MPP CAGE promoters. Statistical significance was calculated as described in Fig. 4C legend (*P < 0.05; **P < 0.01; ***P < 0.001). (**D**) GREAT analysis of cell-specific SEs.

Super-enhancers (SEs) have been described as clusters of enhancers, involved in the specification of cell identity^8^. By using H3K27ac ChIP-seq data, we defined 755 SEs in HSPC, 513 in EPP and 600 in MPP (Supplementary Table 6 and Fig. 5A), all actively transcribed as previously described^9^. The majority of SEs were cell-specific (Fig. 5B) and showed a moderate enrichment in common and cell-specific TF motifs (Supplementary Fig. 9). Expression of CAGE promoters close to EPP- and MPP- specific SEs tended to be higher than the average expression of the total CAGE promoter population in the same cells, or the expression of the same promoters in other cell types (Fig. 5C). HSPC-specific SEs were associated with genes involved in metabolic processes, RNA processing, and T cell phenotypes, while EPP- and MPP-specific SEs were associated to erythroid and myeloid phenotypes, respectively (Fig. 5D).

### Retroviral scanning defines a sub-population of mostly cell-specific regulatory elements

**Figure 6:**
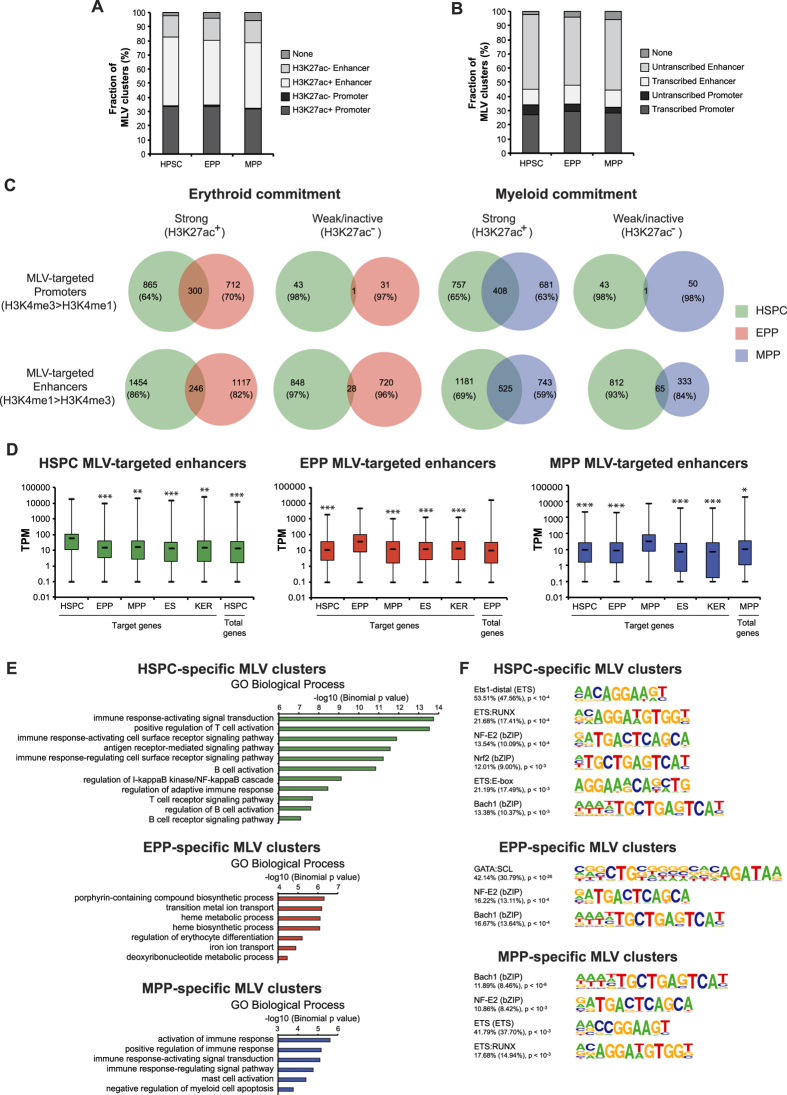
Characterization of MLV-targeted regulatory regions. (**A**,**B**) Fraction of MLV clusters overlapping with epigenetically defined H3K27ac^+^, H3K27ac^−^, transcribed and untranscribed regulatory regions. (**C**) Dynamics of MLV-targeted promoters and enhancers upon HSPC commitment. Venn diagrams show the overlap of MLV-targeted strong (H3K27ac^+^) and weak/inactive (H3K27ac^−^) promoters (H3K4me3 > H3K4me1) and enhancers (H3K4me1 > H3K4me3) identified in HSPC, EPP and MPP. The fraction of non-overlapping HSPC, EPP and MPP regulatory regions hit by MLV is indicated. We defined a total of 1,241 HSPC-specific, 1,998 EPP-specific and 1,833 MPP-specific MLV clusters. (**D**) Distribution of expression levels of CAGE promoters in a ±5 kb interval centered on cell-specific enhancers hit by MLV. As control, expression levels of total HSPC, EPP and MPP CAGE promoters were analyzed. A t-test was performed as described in Fig. 4C legend (*P < 0.05; **P < 0.01; ***P < 0.001). A similar correlation was observed from promoters in a 100-Kb window around the enhancers (data not shown). (**E**) GREAT was used to assign a biological meaning to cell-specific MLV clusters. The analysis showed that MLV is able to target genomic regions involved in cell-specific functions. (**F**) Top enriched TF motifs in cell-specific MLV clusters. HOMER was used to predict TFBS in cell-specific genomic regions targeted by MLV. The frequency of target (background) sequences enriched in TF motifs and p-values are indicated.

We mapped and analyzed the distribution of >27,000 MLV integration sites in each cell population. About 22% of MLV integrations occurred in TSS-proximal regions and the remaining ones were equally distributed in intergenic and intragenic regions (Supplementary Fig. 10A). Statistical comparison with a random dataset identified a total of 3,498, 2,989 and 4,103 integration clusters in HSPC, EPP and MPP, respectively, with a comparable median span of 5.9, 6.0 and 4.8 kb (**see** Supplementary Methods). Virtually all clusters overlapped with epigenetically defined regulatory regions, two-thirds in enhancers and one-third in promoters (Fig. 6A). However, integration clusters targeted only a small fraction of ChIP-defined regulatory regions, i.e., 6% of promoters and 4% of enhancers. Virtually all promoters (97%) and three quarters of the enhancers targeted by MLV integrations were acetylated (compared to ~60 and 15% H3K27ac^+^ non-targeted promoters and enhancers, respectively; Fig. 6A and Supplementary Fig. 10B), and most of the targeted promoters were associated with CAGE transcripts (compared to ~55% transcribed non-targeted promoters; Fig. 6B and Supplementary Fig. 10C). Strikingly, 10 to 13% of the MLV integrations targeted transcribed enhancers, which represented <1% of the total enhancer population (Fig. 6B and Supplementary Table 5). In addition, MLV clusters targeted SEs at a significantly higher frequency compared to the fraction of total active enhancers (53 *vs*. 12%, 57 *vs*. 11% and 73 *vs*. 17% in HSPC, EPP and MPP, respectively, p < 0.0001).

Differently from CAGE and epigenetically defined promoters, only a small proportion of the MLV-targeted promoters were shared between HSPC and their EPP and MPP progeny (Fig. 6C). Likewise, the majority of the MLV-targeted enhancers was cell-specific (Fig. 6C). We observed a higher tendency of weak/inactive regulatory regions to show cell-specificity compared to strong ones (Fig. 6C). For each cell type, the expression level of CAGE promoters flanking cell-specific, MLV-targeted strong enhancers was significantly higher than the average values of the total CAGE promoters in the same cells, or the expression of the same promoters in other cell types (Fig. 6D), indicating that MLV-defined regions are *bona fide* cell-specific and developmentally regulated enhancers. HSPC-specific, MLV-targeted enhancers were associated with genes expressed in hematopoietic organ development and in the immune system, while EPP-specific enhancers were associated to erythrocyte differentiation and MPP-specific enhancers to regulation of the immune system and leukocyte differentiation (Fig. 6E and Supplementary Fig. 11A). In addition, genes flanking cell-specific MLV-targeted enhancers are functionally linked in cell-specific pathways (Supplementary Fig. 11B). The same regions were moderately enriched in hematopoietic TF motifs (Fig. 6F). Comparison with TF ChIP-seq datasets showed that a considerable fraction of MLV cell-specific clusters was targeted by HSPC-, EPP- and MPP-related TFs (Supplementary Fig. 12).

**Figure 7:**
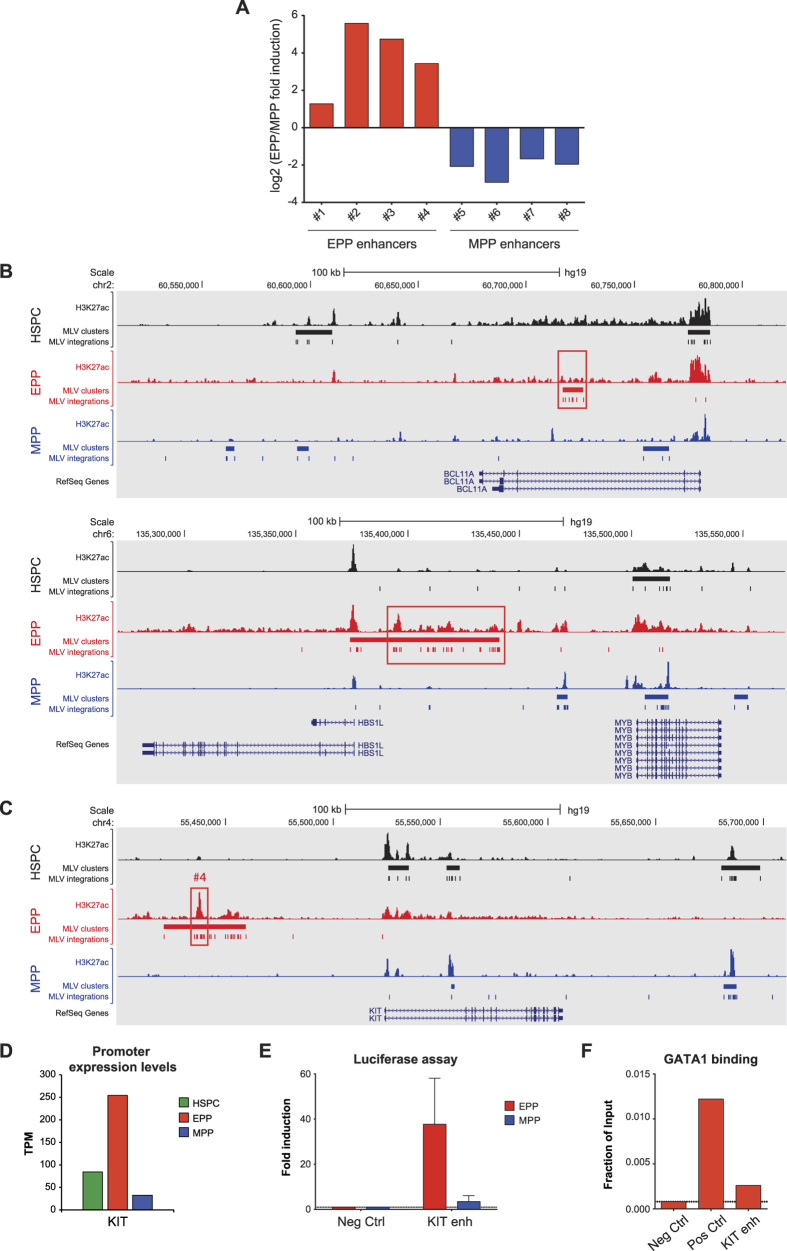
Cell-specific regulatory regions targeted by MLV. (**A**) Validation of putative regulatory elements in hematopoietic primary cells. 8 potential EPP and MPP enhancer elements hit by MLV were cloned by PCR, inserted upstream of a basal promoter and transfected in EPP and MPP. Luciferase activity was quantitated after 18 hr. Fold induction relative to a negative control region was calculated. The log2 of the ratio between EPP and MPP fold induction for each enhancer is shown. All the putative enhancers were able to induce the transcription of the reporter gene in a cell-specific fashion. (**B**) MLV targets erythroid-specific regulatory regions in EPP. MLV clusters and integrations targeting the intronic enhancer of the *BCL11A* gene (upper panel) and the *HBS1L-MYB* intergenic region containing *MYB* enhancers (lower panel) are highlighted with red boxes. (**C**) Differential MLV integration preferences in HSPC, EPP and MPP inside the KIT locus. The erythroid-specific KIT enhancer (#4) is highlighted with a red box. (**D**) CAGE expression levels of the KIT promoter in HSPC, EPP and MPP. (**E**) Fold luciferase induction of the erythroid-specific KIT enhancer (#4) compared to a negative control region (37- and 3.5-fold luciferase induction in EPP and MPP, respectively). (**F**) The erythroid master regulator GATA1 binds the erythroid-specific KIT enhancer. ChIP assay was performed in EPP to analyze GATA1 binding to the KIT enhancer (#4). Two genomic regions were used as negative and positive controls for GATA1 binding, respectively (Neg Ctrl and Pos Ctrl).

To determine whether the putative enhancers identified by combining ChIP-seq and retroviral scanning have transcriptional activity in a functional assay, we tested 8 MLV-targeted erythroid and myeloid enhancers in a reporter assay in EPP and MPP respectively. As expected, erythroid-specific MLV-targeted regions had higher activity in EPP than in MPP and *vice versa* (Fig. 7A), confirming that MLV identifies cell-specific enhancers, possibly controlling the expression of nearby genes. As examples, MLV was able to target known cell-specific regulatory regions, such as the intronic enhancer of the *BCL11A* gene^46^ and the *HBS1L-MYB* intergenic region containing erythroid-specific *MYB* enhancers^47^ (Fig. 7B and Supplementary Fig. 13). MLV scanning identified also novel enhancers in a cell-specific fashion (Supplementary Fig. 13), such as the integration clusters mapping to different regions of the *KIT* locus in HSPC, EPP and MPP (Fig. 7C). These clusters most likely identify enhancers used to exert a lineage-specific control of the locus during hematopoietic differentiation (Fig. 7D and Supplementary Fig. 14), such as the erythroid-specific enhancer #4, which is primarily active in EPP (Fig. 7E) and is targeted by the erythroid master regulator GATA1 (Fig. 7F).

## Discussion

Human hematopoiesis is a well-characterized system in which HSCs give rise to a multilineage progeny through progressive commitment and differentiation of a hierarchy of lineage-restricted progenitors. The expression of specific surface markers allows isolating progenitors and their progeny, and subjecting them to genome-wide analysis of transcriptomes and regulatory elements. Epigenetically defined enhancers and promoters and transcriptional profiles have been reported for human HSPC^9^,^18^ and mature precursors^5^,^18^,^19^,^20^,^21^,^22^,^23^,^24^. In the present study, we attempted to define the chromatin and transcriptional dynamics underlying human hematopoietic lineage commitment in early erythroid and myeloid progenitors. Gene expression analyses and clonogenic assays showed that our EPP and MPP populations represent earlier stages of erythroid and myeloid differentiation compared to the mature cells analyzed in previous studies^18^,^19^,^20^,^21^,^48^.

HSPC and their committed progeny shared most of the promoters and transcripts, suggesting that transcriptional states are largely maintained in early hematopoietic differentiation and only a relatively small number of DU promoters determine progenitor identity. The differentially regulated fraction of promoters transcribe for the most part DE genes and are enriched in binding sites for TFs essential for hematopoietic development. Interestingly, we identified only 78 promoters expressed exclusively in HSPC and down-regulated in both EPP and MPP, while the remaining down-regulated promoters remained expressed in one of the two lineages. On the contrary, >95% of the promoters up-regulated in EPP and MPP were lineage-specific. These data indicate the existence of very few strictly HSPC-specific promoters and factors maintaining multilineage potential, and that lineage commitment is exerted by up-regulation of a few hundred promoters, including those driving the expression of known lineage-specific master TFs, as well as TFs and chromatin modifiers not previously associated with the erythroid and myeloid commitment.

One third of the DU promoters were novel and mapped to intragenic or intergenic regions. Overall, we discovered 577 cell-specific novel promoters, driving the expression of potentially regulatory ncRNAs. These promoters were enriched in cell-specific TFBS and were surrounded by protein-coding, cell-specific genes. A high proportion (40%) of unannotated DU CAGE promoters were marked by histone modifications typical of enhancers. These results support the hypothesis that a consistent fraction of novel CAGE promoters drives the expression of eRNAs, possibly involved in the regulation of proximal genes^49^,^50^,^51^,^52^,^53^,^54^ and in the fine tuning of HSPC commitment. Moreover, a significant fraction of DU CAGE promoters overlapped with transposable elements, which play a role in developmental gene regulation and specification of cell fate^55^,^56^,^57^,^58^.

Analysis of histone modification signatures allowed the identification of >49,000 putative enhancers in each cell population, indicating that most promoters interact with multiple enhancers. Differently from promoters, enhancers consistently changed upon commitment: >50% of the active enhancers and SEs mapped in EPP and MPP were not shared with HSPC, and 60 to 80% of the active enhancers and SEs mapped in HSPC disappeared in EPP and MPP. These data indicate that enhancers are dramatically redefined during lineage commitment, and that differential enhancer usage is responsible for the differential regulation of promoter activity underlying lineage restriction. Activation of the set of lineage-specific enhancers is most likely responsible for both activation of lineage-specific promoters and fine tuning of the non-specific ones.

To provide additional clues to the genomic regions defining cell identity, we exploited the integration properties of MLV, a retrovirus that targets chromatin regions, epigenetically marked as active promoters and enhancers^35^,^37^. This preference is the consequence of the direct binding of the MLV integrase to BRD4, which tether the virus to acetylated histones^38^,^39^,^40^,^41^. By using MLV as a biological scanner, we mapped ~10,000 regulatory regions, representing a small fraction of the ChIP-defined promoters and enhancers (~5%). Acetylated enhancers represented the major target of MLV integrations, as well as transcriptionally active, acetylated promoters. In particular, MLV targeted at high frequency CAGE-defined transcribed enhancers (~40%) and SEs (>50%), enriched in H3K27 acetylation and BRD4 binding sites. Interestingly, MLV scanning identifies at high frequency cell-specific regulatory elements and differentially expressed promoters: while most of the active promoters are shared by HSPC and committed progenitors, MLV clusters mapped preferentially their cell-specific fraction. Moreover, the expression level of transcripts driven by promoters close to MLV-targeted enhancers was significantly higher in each cell type compared to the others, indicating that MLV-defined regulatory regions are *bona fide* cell-specific enhancers. The analysis of a larger dataset of integrations (>1 million) in HSPC^37^ showed that the same genomic regions and genes related to HSPC cell identity were highly targeted by MLV (data not shown). Chromosome conformation capture combined with immunofluorescence experiments will be performed to investigate whether MLV is recruited to transcription factories, where enhancers are juxtaposed to promoters of co-expressed genes^59^, and eRNAs expressed in response to RNA PolII transcription.

Overall, through a combination of transcriptional, epigenetic and MLV signatures, this study provides a genome-wide description of chromatin and transcriptional dynamics and a collection of unannotated regulatory elements differentially used during commitment of human hematopoietic progenitors. In particular, the MLV integration preferences provide a biological scanning function for regulatory elements specifically active during cell state transition and involved in the specification of cell identity.

## Methods

### Cell culture and purification

We obtained human cord blood from healthy donors. Informed consent was obtained from all subjects. All experiments were performed in accordance with the Declaration of Helsinki (TIGET01 protocol). San Raffaele Scientific Institute Ethical Committee approved this study. Culture conditions and purification are described in Supplementary Methods.

### Gene expression profiling and microarray analysis

We determined the transcriptional profiles of multipotent and lineage–committed progenitors using Affymetrix HG-U133 Plus 2.0 GeneChip arrays (3 biological replicates for each population) (Affymetrix, Santa Clara, CA). We performed quality controls in R using the Bioconductor *AffyQControl* package. All arrays in the dataset have good and reproducible quality metrics according to standard guidelines. To convert CEL file fluorescence signals to log2 expression values, we used the robust multi-array average procedure RMA of the Bioconductor *affy* package and the HG-U133 Plus 2.0 custom Chip Definition Files (CDF) based on GeneAnnot^60^. We used Principal Component Analysis (PCA) coded by the *prcomp* function of the R *stats* package to verify the reproducibility of the 3 biological replicates. We used DNA-Chip Analyzer (dChip) software^61^,^62^ to identify differentially expressed genes with fold-change >2, p-value < 0.05, and False Discovery Rate < 5%. In dChip, the False Discovery Rate (FDR) has been estimated permuting the sample labels randomly and computing the number of genes that satisfied the comparison criteria when applied to the randomly composed groups, i.e., quantifying the number of false positives (see http://www.hsph.harvard.edu/cli/complab/dchip/ for details).

### CAGE

We extracted RNA from multipotent and lineage-committed progenitors obtained from a pool of 3 donors. DNAFORM Inc. (Japan) performed DeepCAGE library preparation and data analysis. We defined TSSs by summing the weighted number of CAGE tags at each genome position. Then we clustered TSSs to define promoters if they were within 20 bp of each other. Using a custom R-script, we annotated promoters using publicly available data sets. We validated CAGE data by qRT-PCR. The details of promoter construction and annotation, statistical analyses and qRT-PCR are described in Supplementary Methods.

### ChIP-seq

We performed ChIP-seq for H3K4me3, H3K4me1 and H3K27ac using a pool of 3 donors. ChIP library preparation and sequencing are described in Supplementary Methods. To identify promoters and enhancers, we developed a custom R-workflow that analyzes H3K4me3 and H3K4me1 islands generated by SICER^63^. We used H3K27ac to define active regions. Super-enhancers were defined using ROSE code^8^,^64^. Detailed information is available in Supplementary Methods.

### Retroviral scanning

We transduced EPP and MPP obtained from a pool of 3 donors, mapped MLV integration sites by LM-PCR and pyrosequencing, and defined clusters of recurrent integrations, as previously described^65^. We used luciferase assays to validate putative MLV-targeted enhancers (see Supplementary Methods).

### Bioinformatic analyses

We performed gene functional annotation using Ingenuity Pathways Analysis^66^, DAVID 6.7^67^, and GREAT^68^. We performed TF motif finding using HOMER^22^. Background sequences were automatically selected and weighted to resemble the same GC-content distribution observed in the target sequences. Top enriched motifs were shown. Motifs of TFs not expressed in these cell types, enriched in <5% of the target sequences, or associated with a p-value > 10^−2^ were excluded.

## Additional Information

**Accession codes:** The datasets supporting the results of this article are available in the Gene Expression Omnibus repository under the accession number GSE70677.

**How to cite this article**: Romano, O. *et al.* Transcriptional, epigenetic and retroviral signatures identify regulatory regions involved in hematopoietic lineage commitment. *Sci. Rep.* **6**, 24724; doi: 10.1038/srep24724 (2016).

## Supplementary Material

Supplementary Information
