# Supplementary material for: Transcriptional, epigenetic and retroviral signatures identify regulatory regions involved in hematopoietic lineage commitment
Source: Sci Rep. 2016 Apr 20;6:24724. doi: 10.1038/srep24724 (PMC4837375; doi:10.1038/srep24724)
Supplement: Supplementary Information [file srep24724-s1.pdf]

SUPPLEMENTARY INFORMATION

Transcriptional, epigenetic and retroviral signatures identify regulatory regions involved in hematopoietic lineage commitment

Oriana Romano, Clelia Peano, Guidantonio Malagoli Tagliazucchi, Luca Petiti, Valentina Poletti, Fabienne Cocchiarella, Ermanno Rizzi, Marco Severgnini, Alessia Cavazza, Claudia Rossi, Pasqualepaolo Pagliaro, Alessandro Ambrosi, Giuliana Ferrari, Silvio Bicciato, Gianluca De Bellis, Fulvio Mavilio, and Annarita Miccio.

Supplementary Figure 1

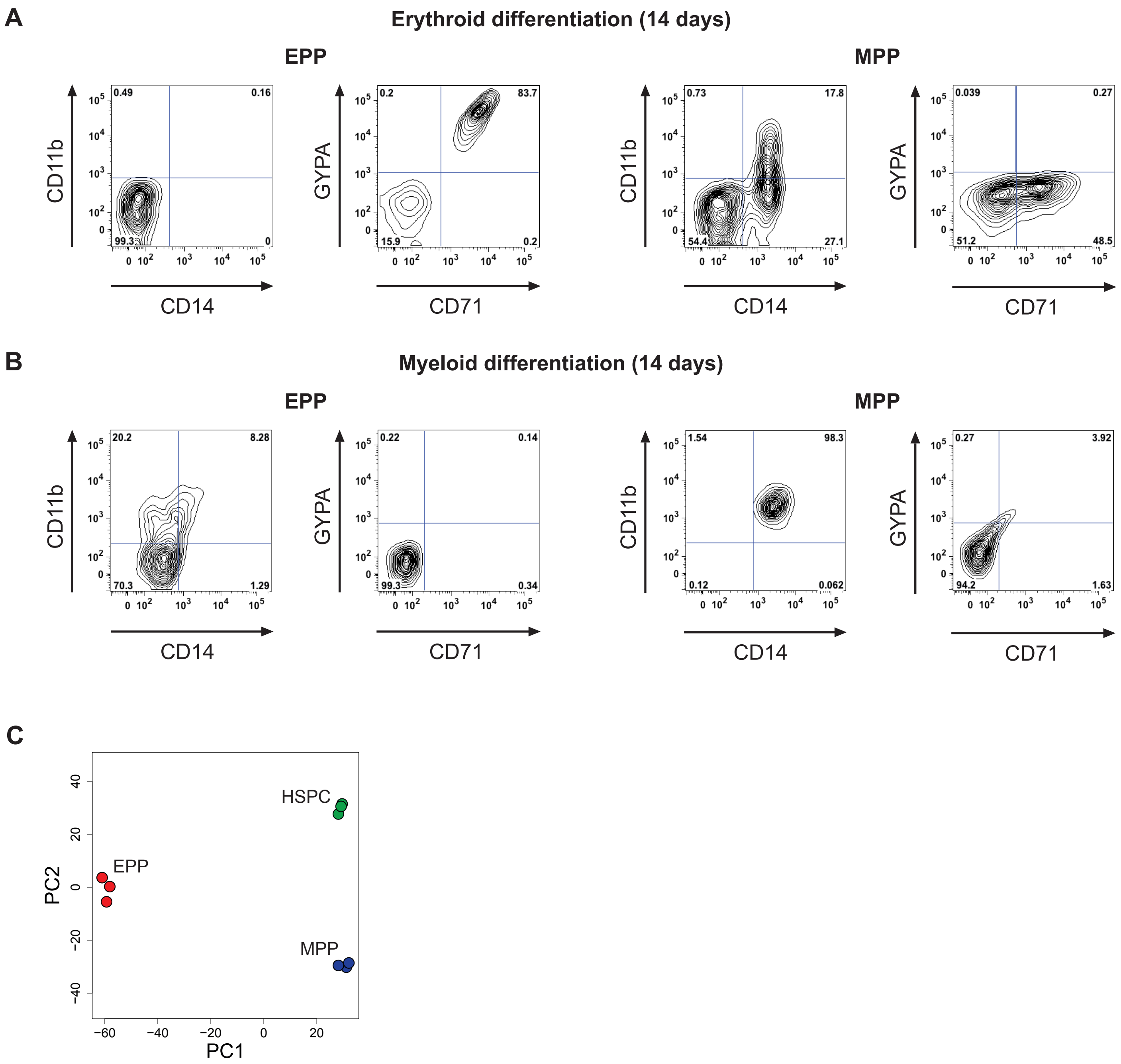

Supplementary Figure 2

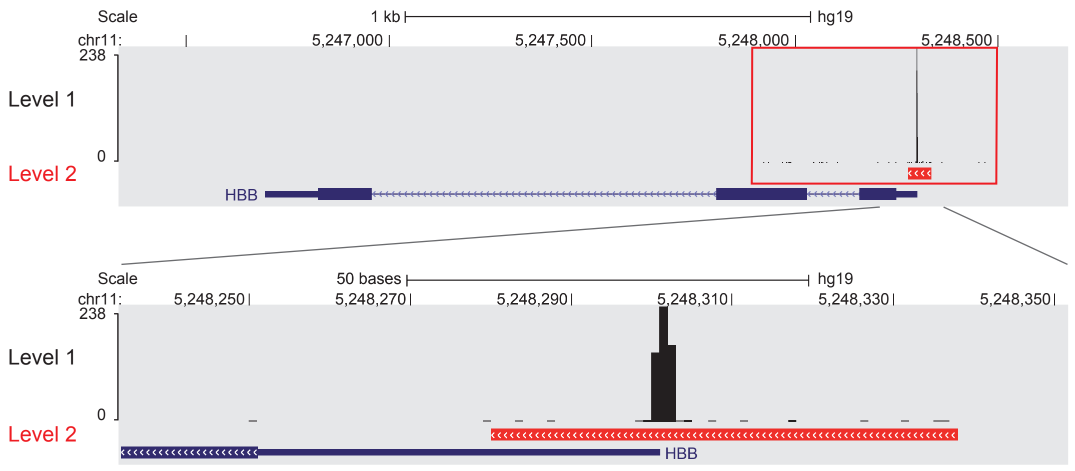

Supplementary Figure 3

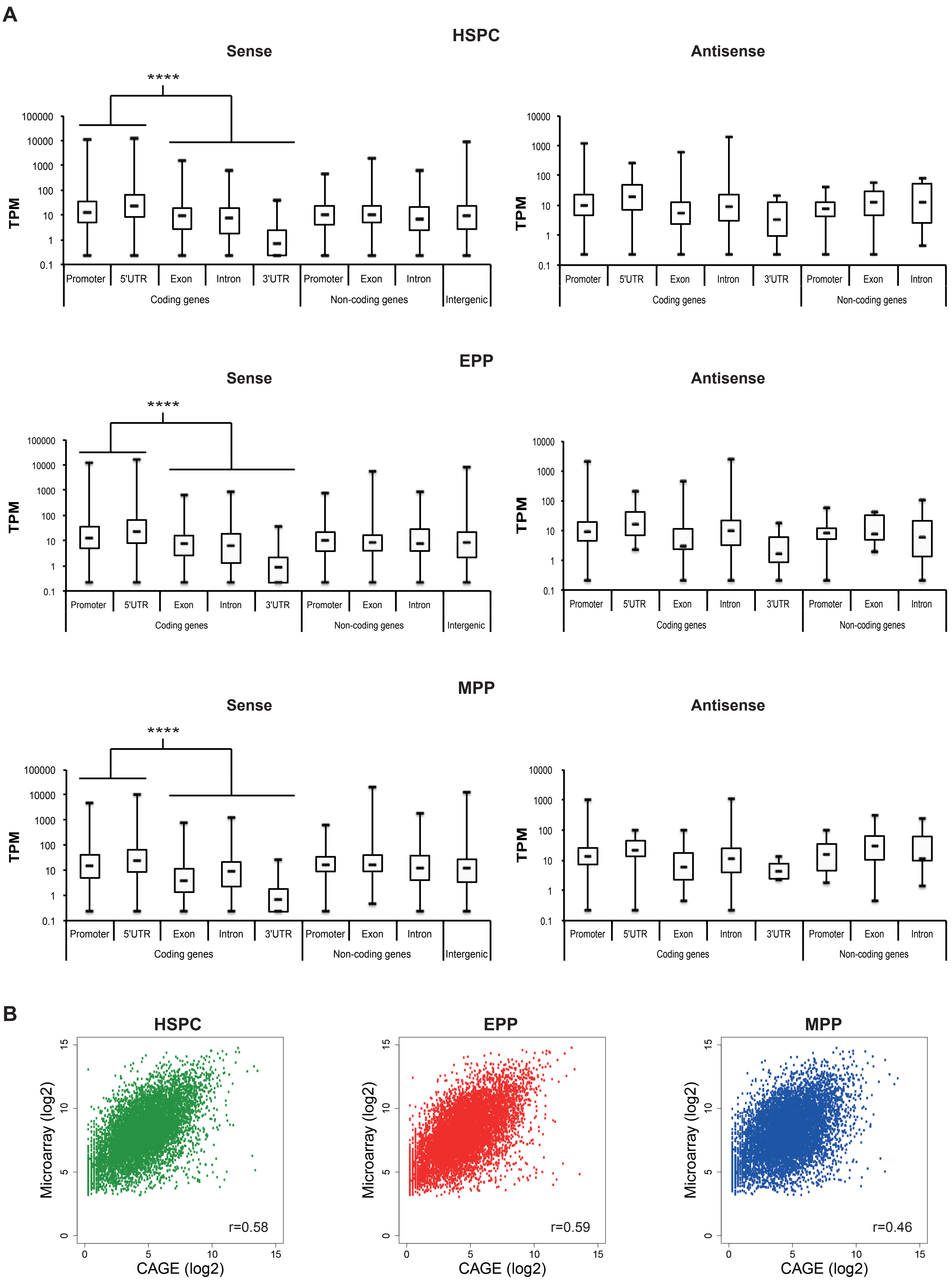

Supplementary Figure 4

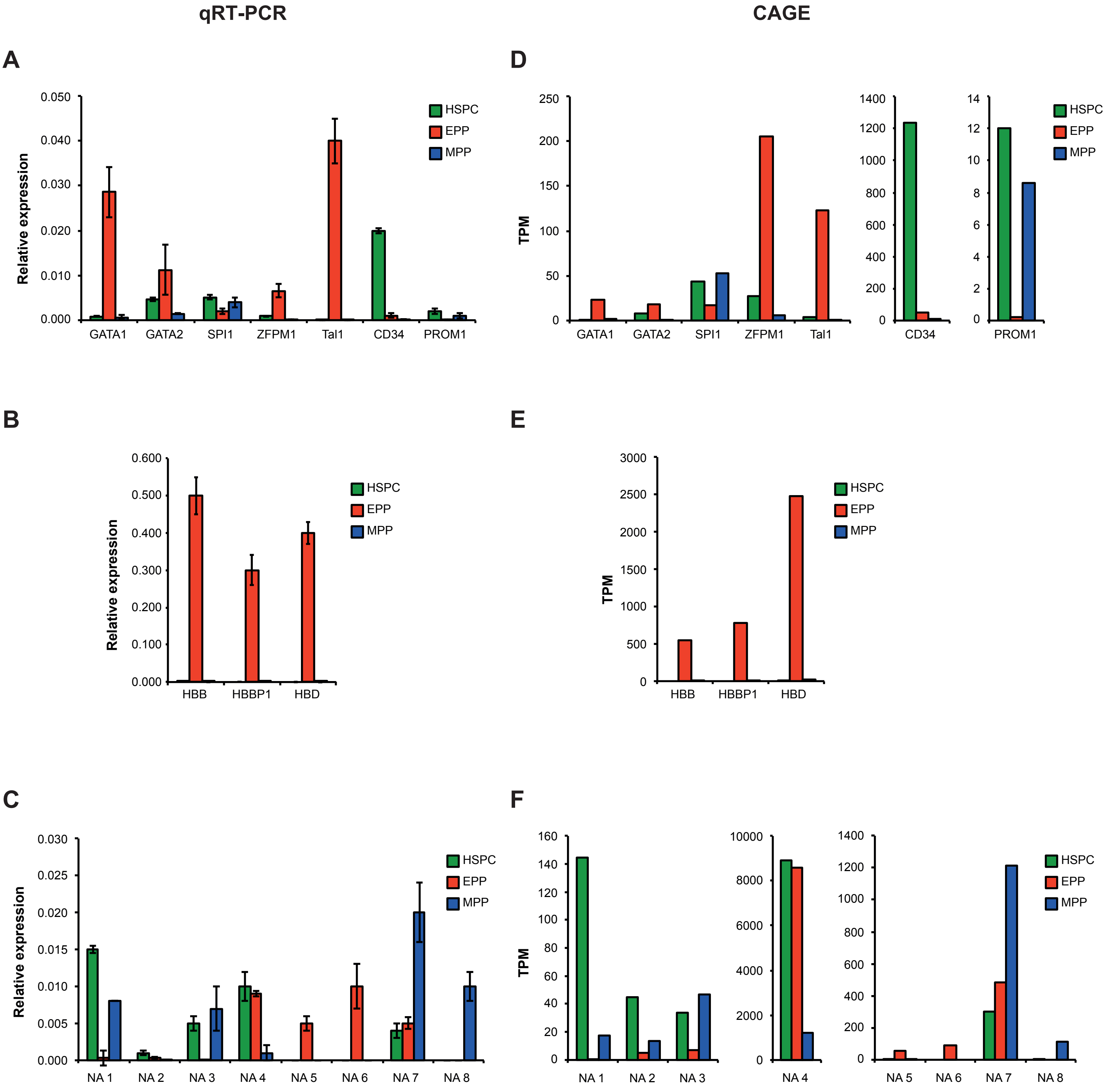

Supplementary Figure 5

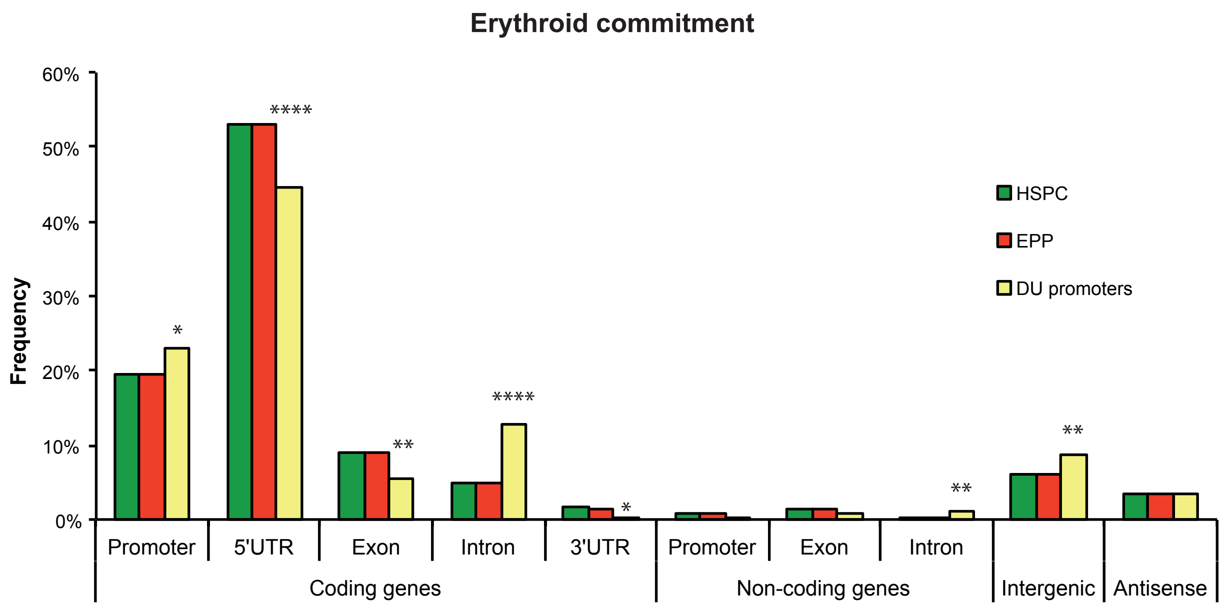

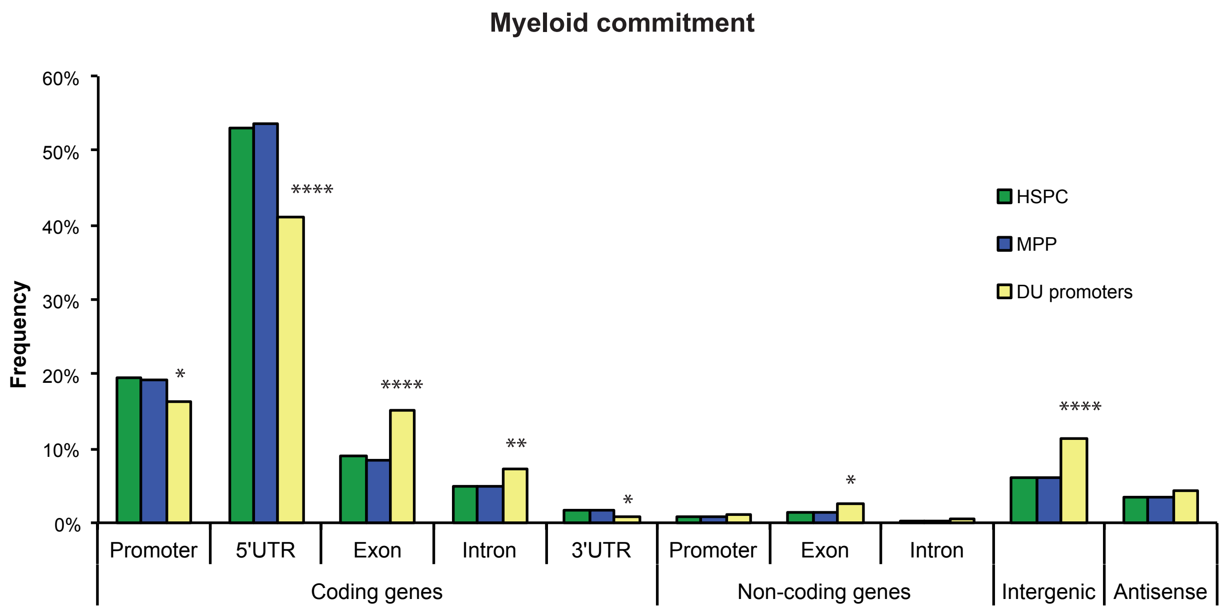

Supplementary Figure 6

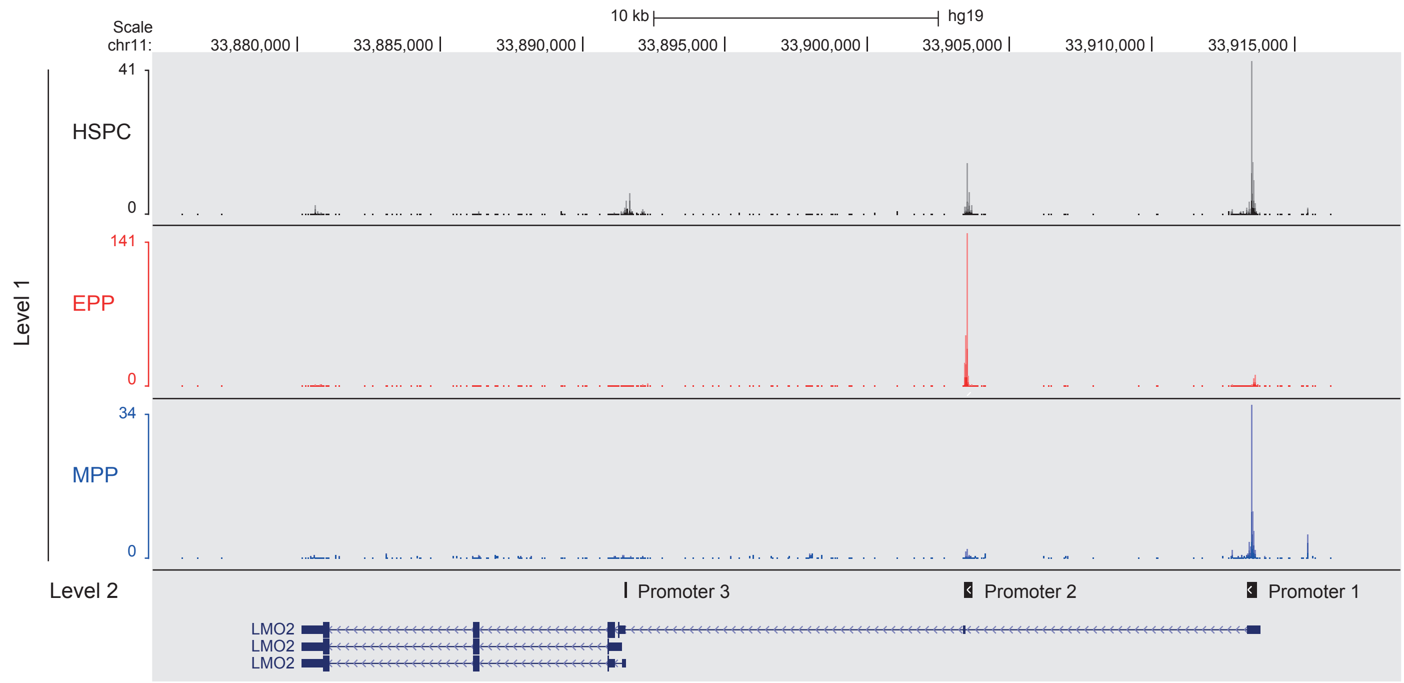

Supplementary Figure 7

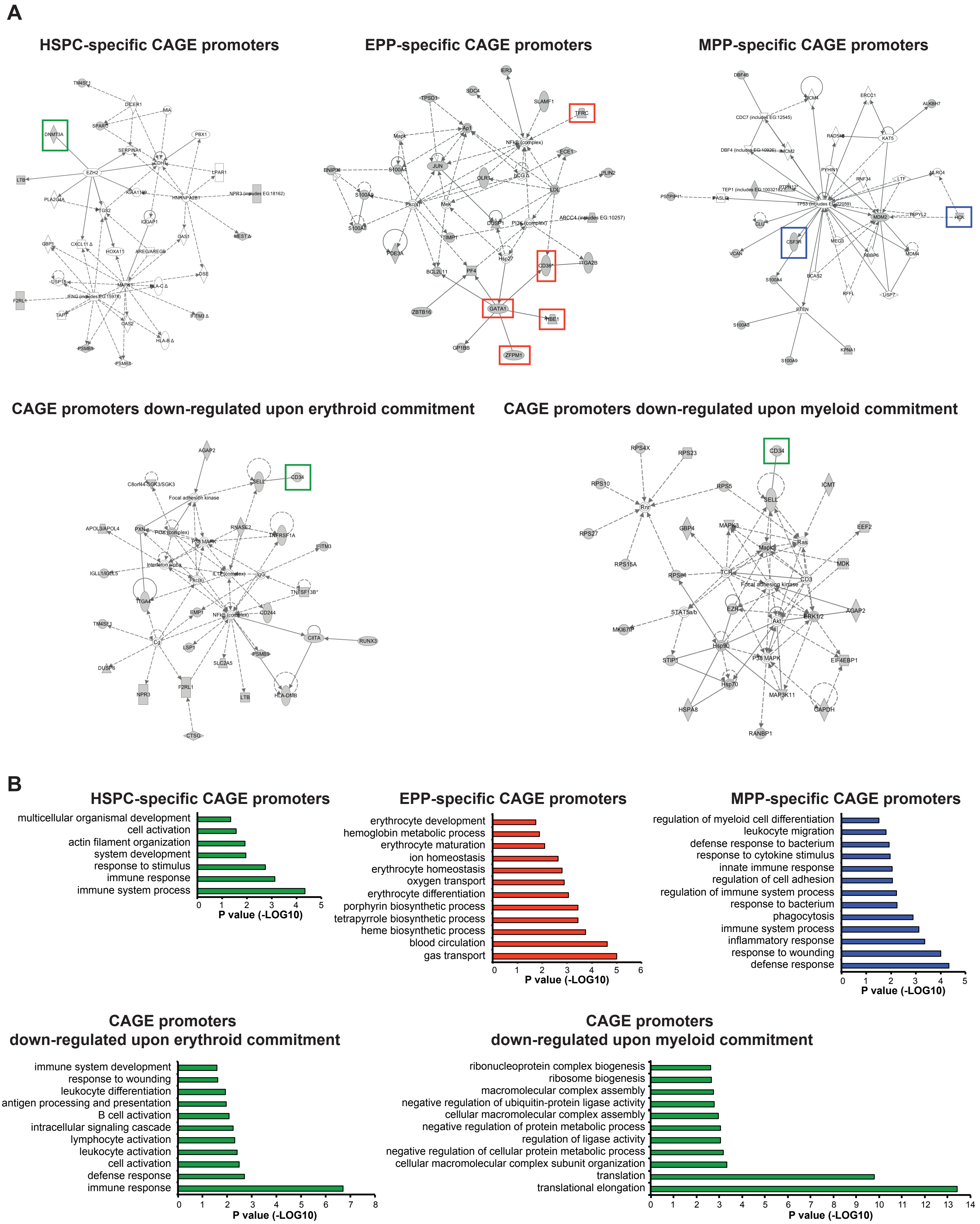

Supplementary Figure 8

HSPC-specific active enhancers

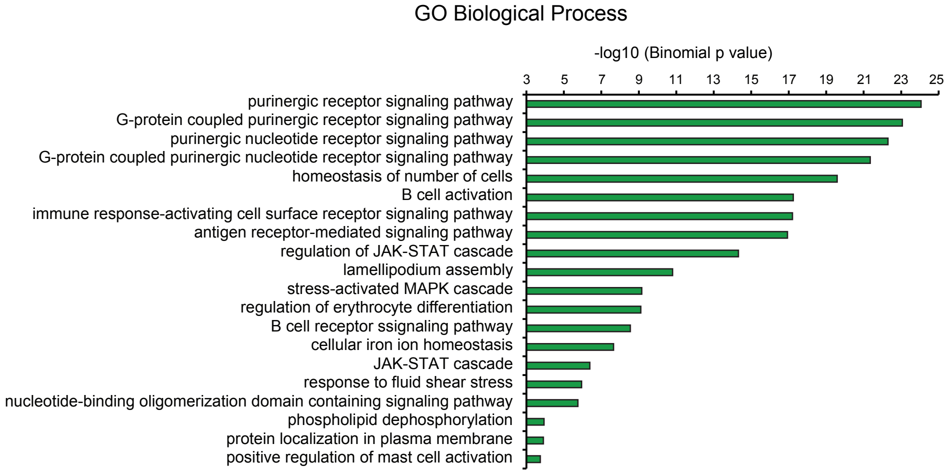

EPP-specific active enhancers

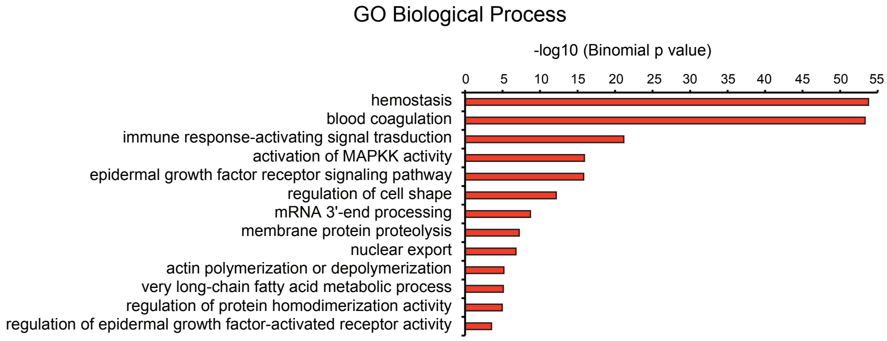

MPP-specific active enhancers

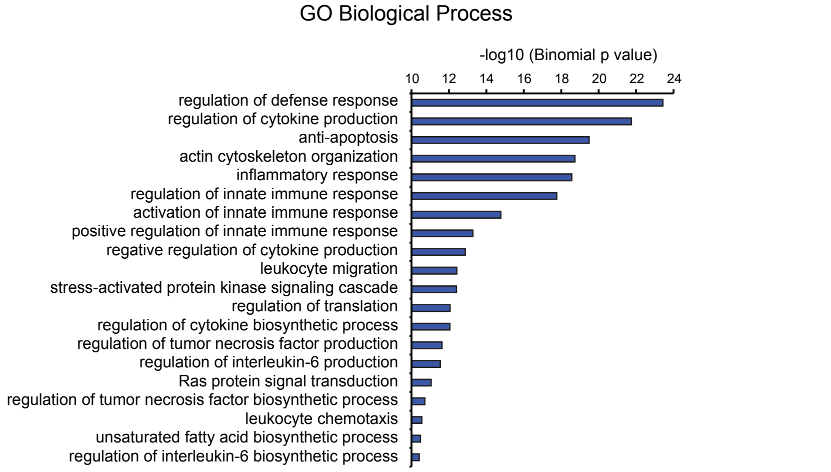

Supplementary Figure 9

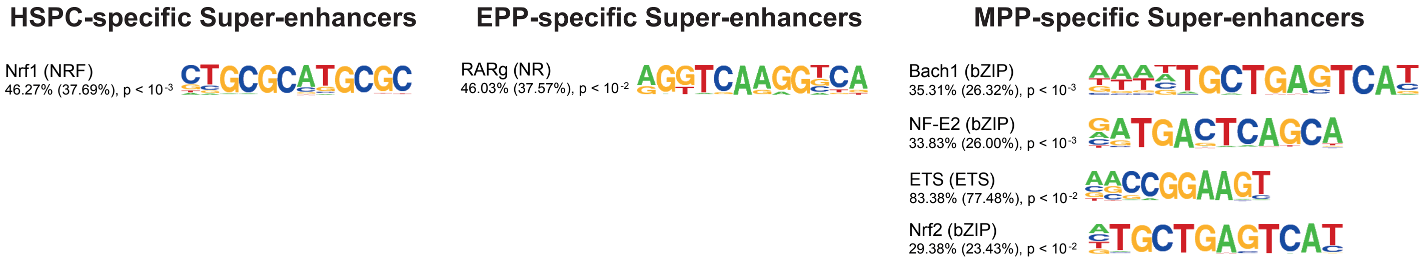

Supplementary Figure 10

A

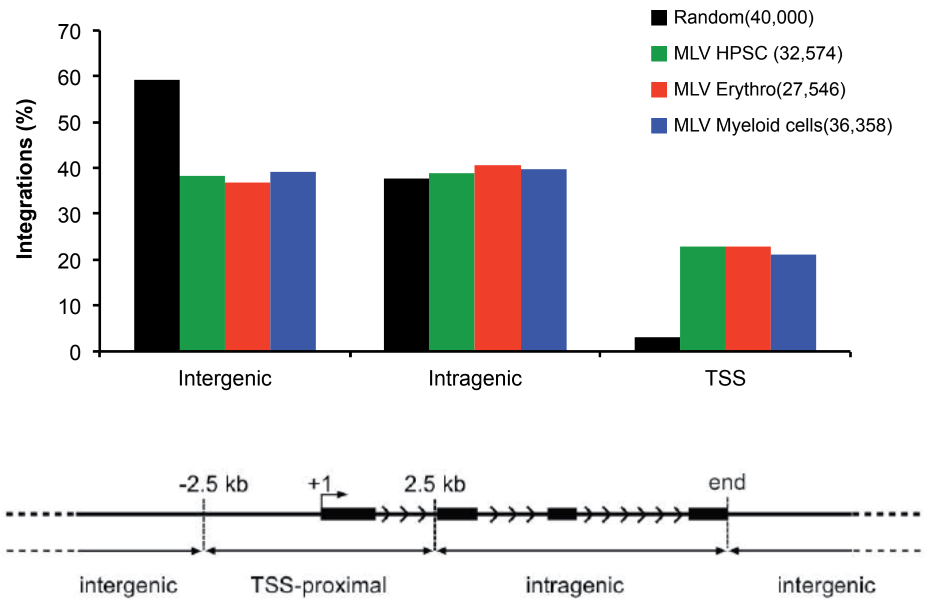

B

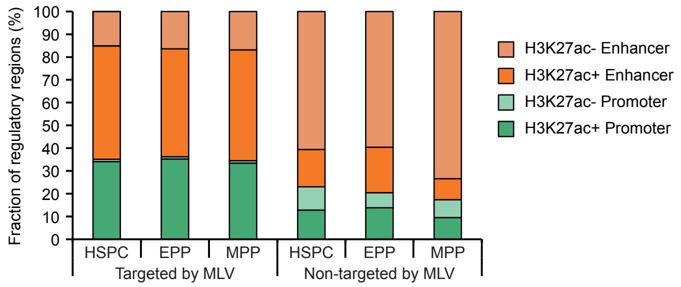

C

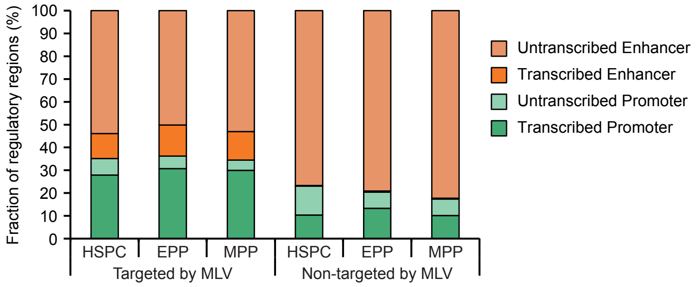

Supplementary Figure 11

A

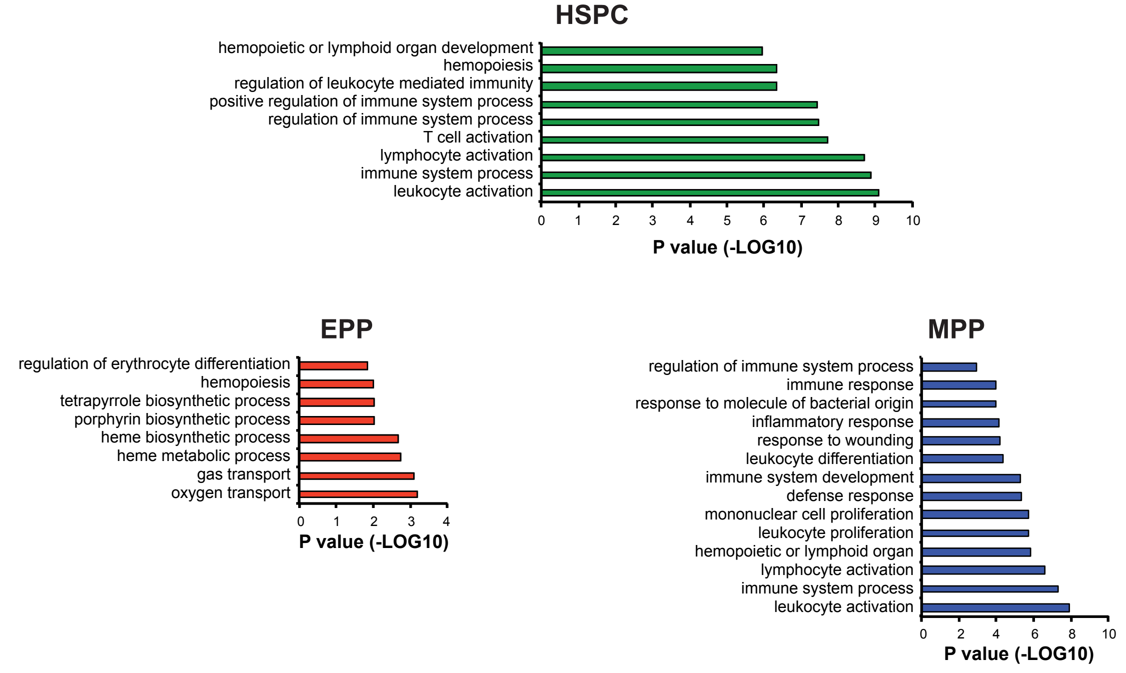

B

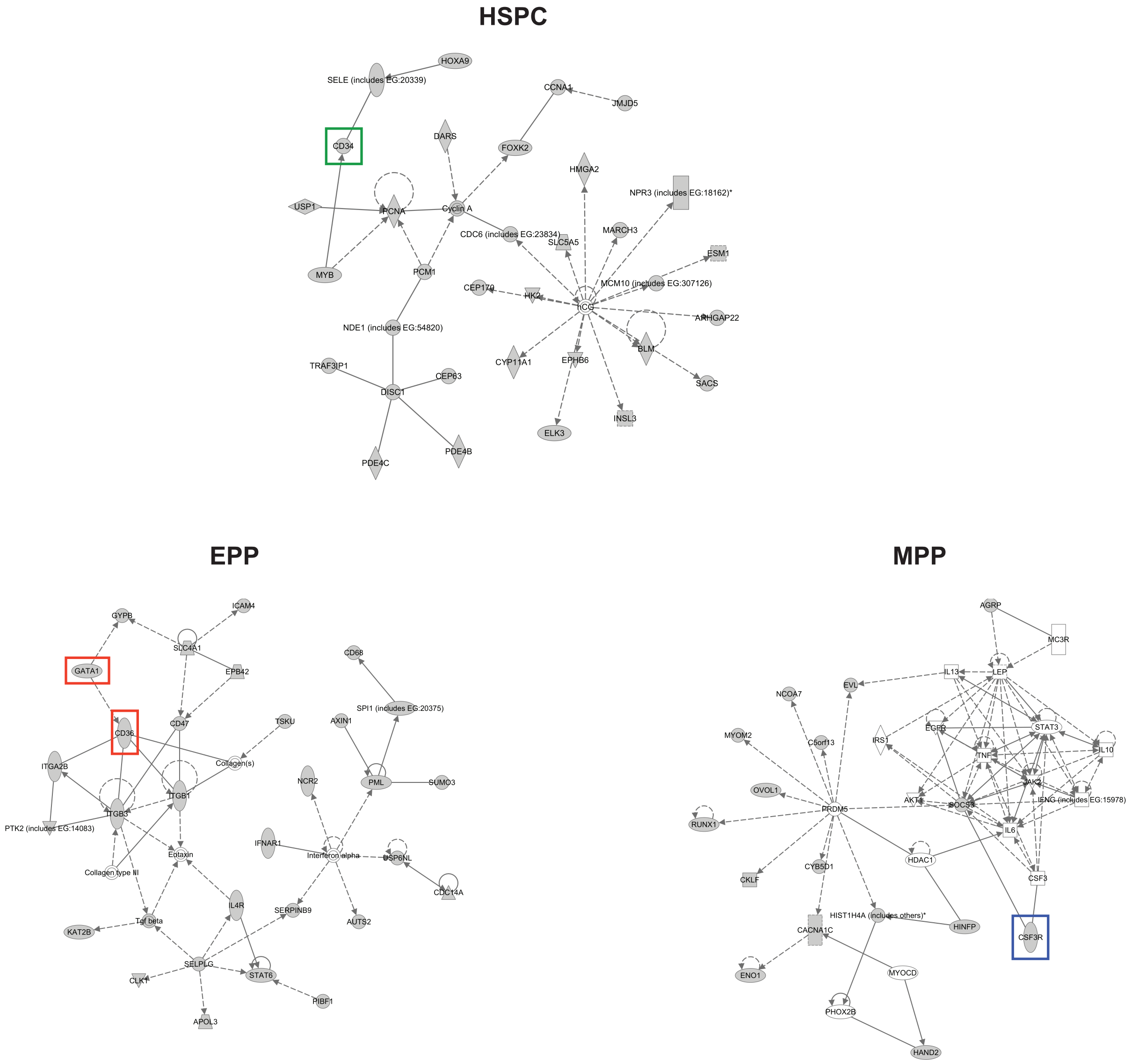

Supplementary Figure 12

A

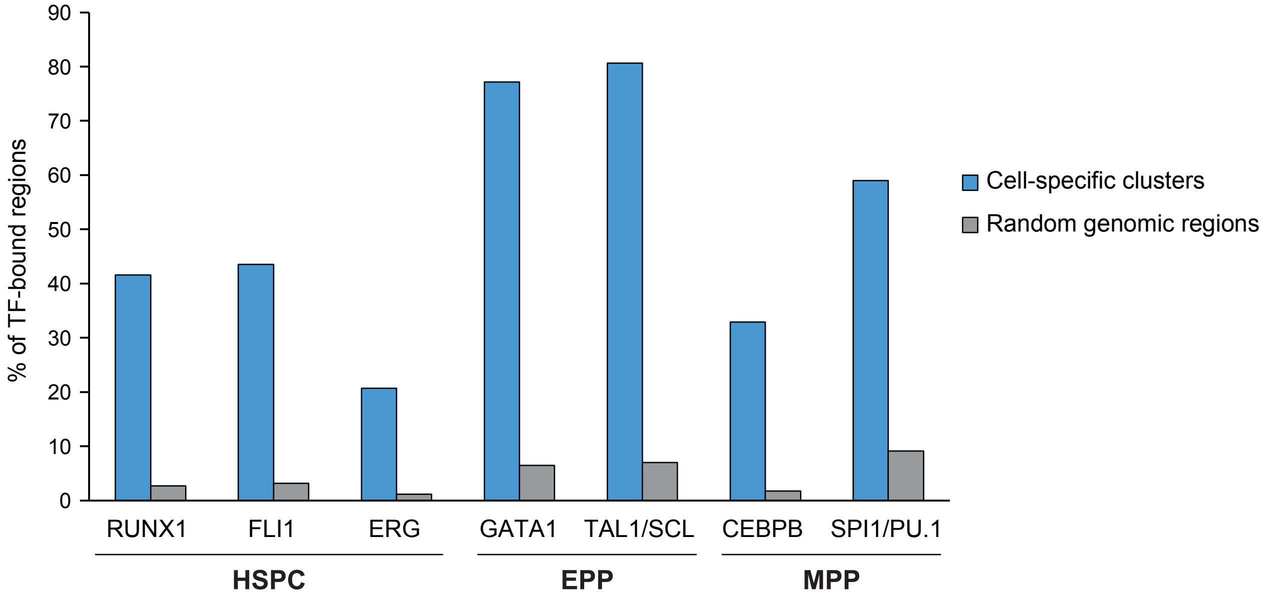

Supplementary Figure 13

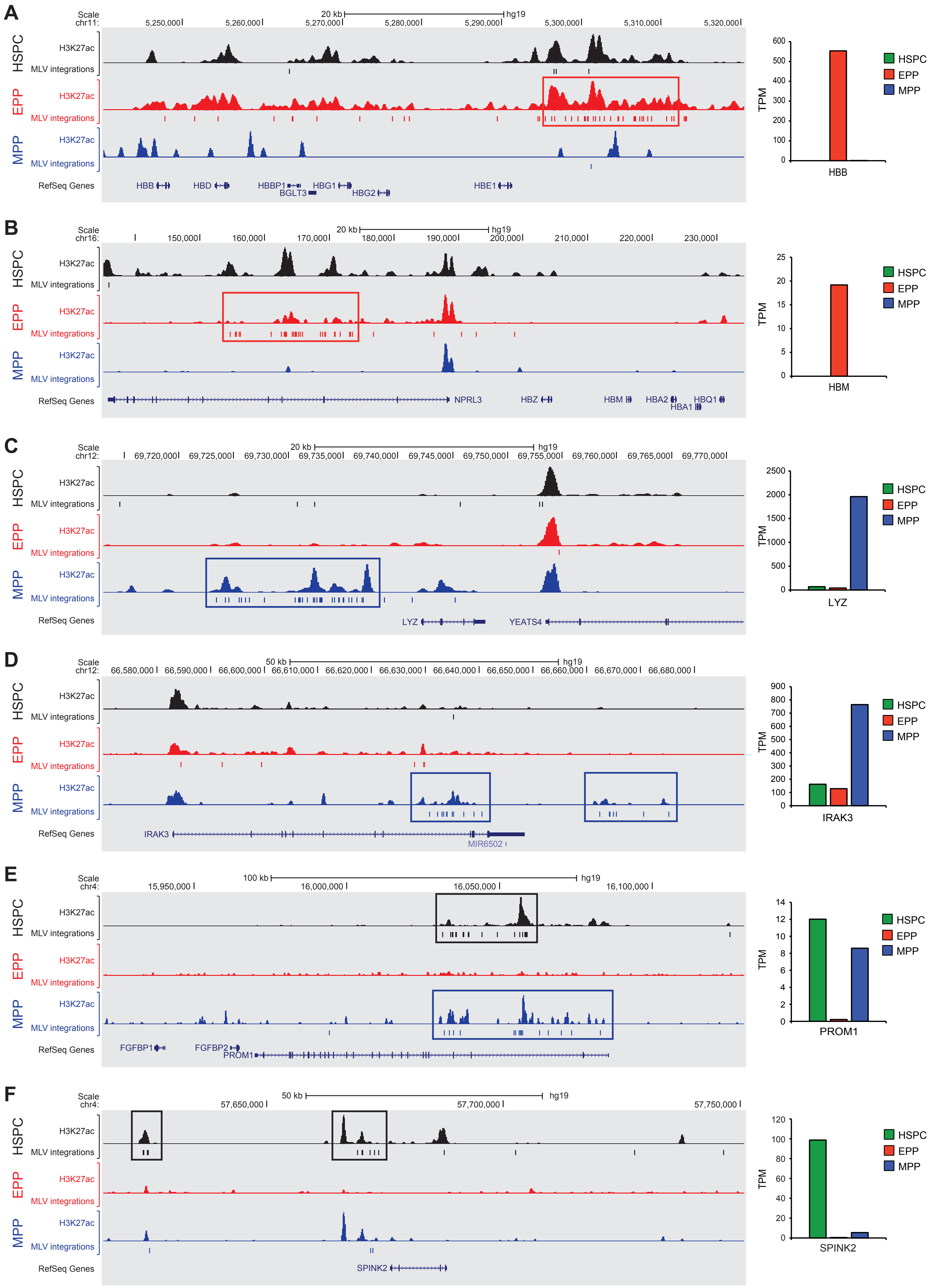

Supplementary Figure 14

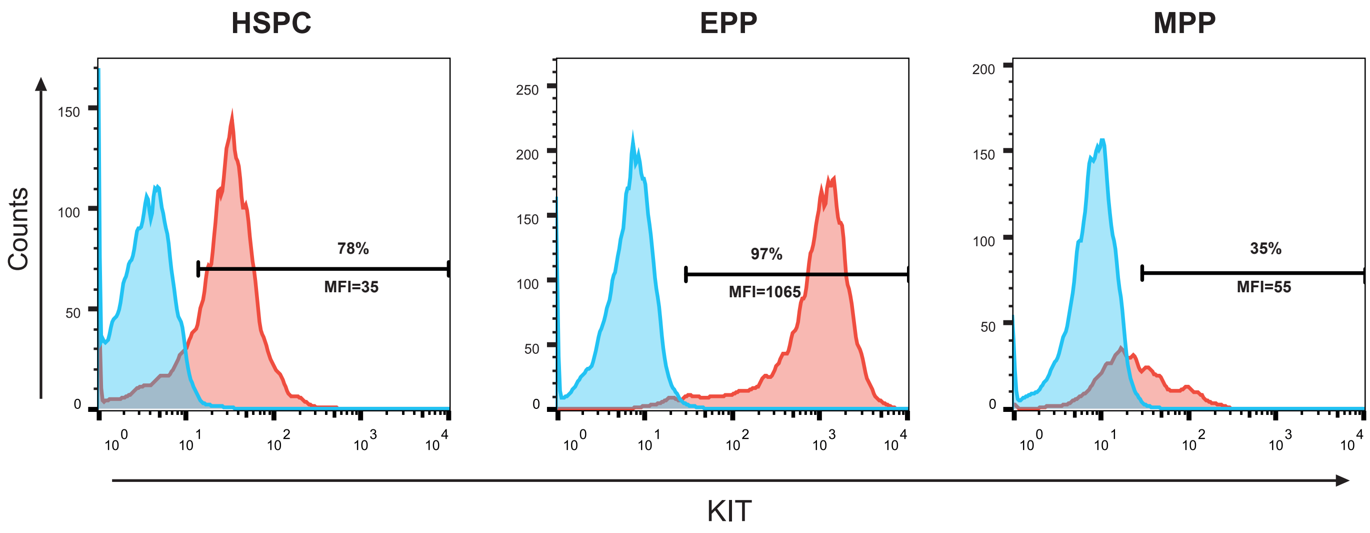

SUPPLEMENTAL FIGURE LEGENDS

Supplemental Figure 1. Terminal erythroid and myeloid differentiation of EPP and MPP. FACS staining of EPP and MPP in liquid erythroid (A) and myeloid (B) cultures for late markers of erythroid (CD71 and glycophorin A, GYPA) and myeloid (CD11b and CD14) differentiation. (C) Principal Component Analysis (PCA) of microarray HSPC, EPP and MPP gene expression.

Supplemental Figure 2. Human beta globin gene (*HBB*) level 1 and level 2 promoters. >30 TSSs ("level 1 promoters") in a 573-bp region encompassing the 5' UTR and the first and second exons of *HBB* (upper panel, highlighted with a red box) were associated to the *HBB* gene. We defined a 58-bp HBB level 2 CAGE promoter (red bar in both upper and lower panels) containing 16 TSSs (including the 3 mostly used TSSs; lower panel), which were clustered since they were within 20 bp of each other on the same chromosomal strand. The remaining TSSs mapping to the 573-bp region were not clustered to define a CAGE promoter, as their expression is lower then 10 tpm and/or they do not map closely (within 20 bp of each other on the same chromosomal strand).

Supplemental Figure 3. Expression levels of CAGE promoters in HSPC, EPP and MPP. (A) CAGE TSSs were mapped to regions annotated as promoters, 5'UTR, introns, exons and 3'UTR of coding and ncRNA, or to intergenic regions (in sense or antisense orientation). The distribution of expression levels is shown for each category of CAGE TSSs. The median expression value is significantly lower for CAGE TSSs overlapping with introns, exons and 3'UTR of coding genes (24, 22 and 2 TPM in HSPC; 19, 23 and 2 TPM in EPP and 17, 32 and 2 TPM in MPP) compared to TSSs mapping to the promoter region of coding RNA (65 and 83 TPM in HSPC; 69 and 81 in EPP and 55 and 77 in MPP; p-value <0.0001). Transcripts originating from ncRNA promoters were slightly less abundant (22 TPM in HSPC, 31 in EPP and 41 in MPP) than those arising from promoters and 5' UTRs of coding genes (65 and 83 TPM in HSPC, 69 and 81 in EPP and 55 and 77 in MPP). Expression of TSSs associated with the antisense strand of promoters and 5'UTR of coding transcripts (38 and 43 TPM in HSPC; 47 and 33 TPM in EPP and 39 and 32 TPM in MPP) tended to be lower than those mapping to the sense strand (65 and 83 TPM in HSPC; 69 and 81 in EPP and 55 and 77 in MPP). (B) Scatterplots of log2 transformed expression values detected by Affymetrix microarray and CAGE, in HSPC, EPP and MPP. Pearson r correlation coefficients are indicated.

Supplemental Figure 4. Validation of DU CAGE promoters. (A-C) RNA levels of annotated (A and B) and unannotated (NA; panel C) CAGE promoters were measured by real-time PCR in HSPC, EPP and MPP. All samples were normalized to beta-actin. Bars indicate standard errors of the means (n =3 biological replicates). CAGE values are indicated in panels D, E and F.

Supplemental Figure 5. Genomic distribution of CAGE TSSs associated with DU promoters. CAGE TSSs were mapped as described in legend to Figure 2. The genomic distribution of total CAGE TSSs (HSPC, EPP and MPP) and TSSs associated with DU promoters was significantly different among the different categories (*P<0.05; **P<0.01; ****P<0.0001).

Supplemental Figure 6. Example of alternative promoter usage. Three promoters of *LMO2* gene were differentially used in HSPC, EPP and MPP (Promoter 1, 2 and 3). Promoter 2 was not identified previously. Promoter 1 is mainly active in HSPC and MPP, Promoter 2 is virtually inactive in MPP and expressed at 8-fold-higher levels in EPP compared to HSPC. Promoter 3 is HSPC-specific. The different promoters and their expression level are shown.

Supplemental Figure 7. Functional annotation of genes associated to cell-specific promoters. (A) Genes associated with HSPC-, EPP- and MPP-specific promoters were functionally linked in cell-specific pathways by using Ingenuity Pathways Analysis. Genes essential for HSPC-, EPP- and MPP-specific functions are highlighted with green, red and blue boxes, respectively. (B) Gene ontology

analysis (DAVID 6.7) of genes associated with HSPC-, EPP- and MPP-specific CAGE promoters (EASE score ≤0.05 after Benjamini correction for multiple testing).

Supplemental Figure 8. Gene ontology analysis of genomic regions associated with epigenetically defined regulatory regions. Gene ontology analysis of cell-specific active enhancers was performed using GREAT.

Supplemental Figure 9. Analysis of transcription factor motifs in HSPC, EPP and MPP SE. Top enriched TF motifs in SE were determined using HOMER. The frequency of target (background) sequences enriched in TF motifs and p-values are indicated.

Supplemental Figure 10. Genomic distribution of MLV insertion sites. (A) MLV integrations were annotated as TSS-proximal when occurring in a ±2.5 kb interval around the TSS of a known gene, intragenic when occurring inside a known gene >2.5 kb from the TSS and intergenic in all other cases. A control dataset of random sites served as control (48). (B-C) Fraction of epigenetically defined regulatory regions, targeted and non-targeted by MLV, identified as H3K27ac$^+$, H3K27ac$^-$, transcribed and untranscribed promoters and enhancers

Supplemental Figure 11. Analysis of MLV-targeted genes. (A) Functional annotation of known genes associated with MLV clusters was computed using DAVID 6.7. (B) IPA network analysis of genes targeted by MLV. HSPC-specific genes are highlighted with green boxes. Red and blue boxes indicate genes essential for erythroid and myeloid cell functions, respectively.

Supplemental Figure 12. Analysis of TF occupancy in MLV cell-specific clusters. ChIP-seq of HSPC-, EPP- and MPP-related TF were used to identify TF-bound cell-specific clusters. We analyzed available ChIP-seq datasets (GSM1097879, GSM1097880, GSM1097884, GSM651547, GSM651545, GSM785495 and GSM785496) using BEDTools. Random genomic regions of comparable size served as control.

Supplemental Figure 13. Cell-specific regulatory regions targeted by MLV. (A-B) The Locus Control Region (LCR) in the β-globin locus and the hypersensitive sites controlling α-globin expression are highly targeted by MLV integrations in EPP. (C-D) MLV targets putative regulatory elements controlling the expression of LYZ (lysozyme) and IRAK3 (interleukin-1 receptor-associated kinase 3) genes, which are up-regulated upon myeloid commitment. (E-F) Potential regulatory elements identified by MLV in the PROM1 (CD133) and SPINK2 (serine protease inhibitors of the Kazal type 2) loci of HSPC. For each putative target gene, CAGE promoter expression levels in HSPC, EPP and MPP are indicated.

Supplemental Figure 14. Analysis of KIT expression in HSPC, EPP and MPP by flow cytometry. FACS analysis of HSPC, EPP and MPP. The percentage of KIT positive cells (red histogram plots) and their median fluorescence intensities (MFI) are indicated. Blue curves represent histogram plots of cells incubated with an isotype-matched control antibody.

Supplemental Table 1. Differentially expressed genes by Affymetrix microarray analysis
Genes up-regulated in EPP.

Gene Symbol	Gene Name	Fold change	P value
HBBP1	hemoglobin, beta pseudogene 1	8.03	0.000001
HBB	hemoglobin, beta	7.42	0.000046
HBE1	hemoglobin, epsilon 1	7.26	0.000506
CD36	CD36 molecule (thrombospondin receptor)	6.73	0.000005
RHAG	Rh-associated glycoprotein	6.32	0.000011
CNRIP1	cannabinoid receptor interacting protein 1	6.28	0.005413
TUBB2A	tubulin, beta 2A	6.2	0.000044
NMU	neuromedin U	6.17	0.001302
XK	X-linked Kx blood group (McLeod syndrome)	6.13	0.000027
ERAF	erythroid associated factor	5.85	0.00064
KLF1	Kruppel-like factor 1 (erythroid)	5.4	0.000265
PF4	platelet factor 4	5.28	0.003868
APOC1	apolipoprotein C-I	5.03	0.000018
EPB42	erythrocyte membrane protein band 4.2	5	0.001048
KCNH2	potassium voltage-gated channel, subfamily H (eag-related), member 2	4.94	0.000124
S100A8	S100 calcium binding protein A8	4.9	0.010992
SPTA1	spectrin, alpha, erythrocytic 1 (elliptocytosis 2)	4.85	0.000068
TMEM56	transmembrane protein 56	4.85	0.002134
HBD	hemoglobin, delta	4.75	0.010117
CA2	carbonic anhydrase II	4.72	0.004686
ITGA2B	integrin, alpha 2b (platelet glycoprotein IIb of IIb/IIIa complex, antigen CD41)	4.49	0.00002
TIMP3	TIMP metallopeptidase inhibitor 3	4.46	0.000087
SLC6A8	solute carrier family 6 (neurotransmitter transporter, creatine), member 8	4.45	0.00658
IRF6	interferon regulatory factor 6	4.37	0.002134
KEL	Kell blood group, metallo-endopeptidase	4.36	0.000011
TSC22D3	TSC22 domain family, member 3	4.35	0.001438
PRG2	proteoglycan 2, bone marrow (natural killer cell activator, eosinophil granule major basic protein)	4.32	0.004047
ANK1	ankyrin 1, erythrocytic	4.28	0.000031
ALAS2	aminolevulinate, delta-, synthase 2	4.27	0.002038
PKLR	pyruvate kinase, liver and RBC	4.24	0.001943
RFESD	Rieske (Fe-S) domain containing	4.18	0.000068
ABCC4	ATP-binding cassette, sub-family C (CFTR/MRP), member 4	4.16	0.000007
PNMT	phenylethanolamine N-methyltransferase	4.14	0.00294
CDH1	cadherin 1, type 1, E-cadherin (epithelial)	4.05	0.000001
DHRS3	dehydrogenase/reductase (SDR family) member 3	4.03	0.004707

GATA1	GATA binding protein 1 (globin transcription factor 1)	4.03	0.000503
BLVRB	biliverdin reductase B (flavin reductase (NADPH))	4.03	0.000495
PROS1	protein S (alpha)	4	0.000575
FAM132B	family with sequence similarity 132, member B	3.99	0.0006
CHST2	carbohydrate (N-acetylglucosamine-6-O) sulfotransferase 2	3.98	0.0002
DDIT4	DNA-damage-inducible transcript 4	3.95	0.000967
YPEL4	yippee-like 4 (Drosophila)	3.94	0.000002
GAD1	glutamate decarboxylase 1 (brain, 67kDa)	3.9	0.003131
ALOX5	arachidonate 5-lipoxygenase	3.87	0.002718
CLC	Charcot-Leyden crystal protein	3.79	0.043636
ICAM4	intercellular adhesion molecule 4 (Landsteiner-Wiener blood group)	3.78	0.000346
MYO1D	myosin ID	3.77	0.00622
SEPT10	septin 10	3.75	0.000025
TRIB2	tribbles homolog 2 (Drosophila)	3.73	0.000017
CD24	CD24 molecule	3.73	0.046025
TSPAN6	tetraspanin 6	3.62	0.000025
PMP22	peripheral myelin protein 22	3.49	0.001247
GYPA	glycophorin A (MNS blood group)	3.47	0.009992
ALAS1	aminolevulinate, delta-, synthase 1	3.46	0.00001
HBZ	hemoglobin, zeta	3.45	0.001038
FAM171A1	family with sequence similarity 171, member A1	3.44	0.002002
ATP7B	ATPase, Cu++ transporting, beta polypeptide	3.44	0.00059
APOE	apolipoprotein E	3.44	0.019903
OSBPL6	oxysterol binding protein-like 6	3.37	0.002182
TUBB1	tubulin, beta 1	3.37	0.007354
JAZF1	JAZF zinc finger 1	3.36	0.000144
PPEF1	protein phosphatase, EF-hand calcium binding domain 1	3.36	0.017919
FAM178B	family with sequence similarity 178, member B	3.35	0.019388
NDFIP2	Nedd4 family interacting protein 2	3.34	0.000012
GNAQ	guanine nucleotide binding protein (G protein), q polypeptide	3.33	0.000058
TMOD1	tropomodulin 1	3.3	0.000143
PKM2	pyruvate kinase, muscle	3.3	0.001942
HES6	hairy and enhancer of split 6 (Drosophila)	3.28	0.001757
ZFPM1	zinc finger protein, multitype 1	3.28	0.000246
LXN	latexin	3.27	0.000968
CLCN4	chloride channel 4	3.27	0.000001
CMTM5	CKLF-like MARVEL transmembrane domain containing 5	3.26	0.00545
LEPR	leptin receptor	3.22	0.002566
HBM	hemoglobin, mu	3.18	0.002604
C2orf88	chromosome 2 open reading frame 88	3.12	0.001122
CBS	cystathionine-beta-synthase	3.07	0.005707
PLK1	polo-like kinase 1 (Drosophila)	3.04	0.00106

ADFP	adipose differentiation-related protein	3.03	0.000006
LOC388588	hypothetical LOC388588	3.01	0.000884
EPB49	erythrocyte membrane protein band 4.9 (dematin)	3.01	0.002203
GFI1B	growth factor independent 1B transcription repressor	3.01	0.000122
G0S2	G0/G1switch 2	3	0.000113
AQP1	aquaporin 1 (Colton blood group)	2.99	0.002404
IL2RG	interleukin 2 receptor, gamma (severe combined immunodeficiency)	2.98	0.00869
NUCB1	nucleobindin 1	2.98	0.000366
IL2RA	interleukin 2 receptor, alpha	2.97	0.000043
CITED2	Cbp/p300-interacting transactivator, with Glu/Asp-rich carboxy-terminal domain, 2	2.94	0.000083
RTN1	reticulon 1	2.92	0.038727
MUC1	mucin 1, cell surface associated	2.91	0.000299
HBQ1	hemoglobin, theta 1	2.9	0.000007
FKBP5	FK506 binding protein 5	2.88	0.000086
MYL4	myosin, light chain 4, alkali; atrial, embryonic	2.87	0.005637
LMNA	lamin A/C	2.86	0.000179
WFDC1	WAP four-disulfide core domain 1	2.85	0.014183
KLHDC8B	kelch domain containing 8B	2.84	0.002207
TUBB2B	tubulin, beta 2B	2.82	0.010122
SLC16A9	solute carrier family 16, member 9 (monocarboxylic acid transporter 9)	2.82	0.04153
LMAN1	lectin, mannose-binding, 1	2.82	0.001399
PBK	PDZ binding kinase	2.8	0.000088
PDLIM1	PDZ and LIM domain 1	2.8	0.000875
PPAP2A	phosphatidic acid phosphatase type 2A	2.79	0.001531
TFR2	transferrin receptor 2	2.79	0.003792
DNAJA4	DnaJ (Hsp40) homolog, subfamily A, member 4	2.79	0.006862
TGM2	transglutaminase 2 (C polypeptide, protein-glutamine-gamma-glutamyltransferase)	2.79	0.011459
FCGR2B	Fc fragment of IgG, low affinity IIb, receptor (CD32)	2.78	0.003071
P4HA2	prolyl 4-hydroxylase, alpha polypeptide II	2.78	0.000507
ITGAM	integrin, alpha M (complement component 3 receptor 3 subunit)	2.78	0.020347
ADD2	adducin 2 (beta)	2.77	0.001392
SH2D2A	SH2 domain protein 2A	2.76	0.0313
RGS16	regulator of G-protein signaling 16	2.75	0.002007
EPS8	epidermal growth factor receptor pathway substrate 8	2.75	0.005551
LOX	lysyl oxidase	2.74	0.030823
GTF2I	general transcription factor II, i	2.71	0.00007
REPS2	RALBP1 associated Eps domain containing 2	2.71	0.000129
BNIP3	BCL2/adenovirus E1B 19kDa interacting protein 3	2.71	0.020271
ABCB6	ATP-binding cassette, sub-family B (MDR/TAP), member 6	2.7	0.000164

FHL2	four and a half LIM domains 2	2.69	0.000124
CAST	calpastatin	2.68	0.000004
FZD3	frizzled homolog 3 (Drosophila)	2.68	0.0015
GSTM3	glutathione S-transferase mu 3 (brain)	2.67	0.022365
NCKAP1	NCK-associated protein 1	2.67	0.000688
DARC	Duffy blood group, chemokine receptor	2.66	0.000131
TRAPPC1	trafficking protein particle complex 1	2.66	0.000743
PLD3	phospholipase D family, member 3	2.66	0.001067
COL18A1	collagen, type XVIII, alpha 1	2.65	0.000027
BCL2L11	BCL2-like 11 (apoptosis facilitator)	2.63	0.0006
ARL4A	ADP-ribosylation factor-like 4A	2.63	0.000777
TLE1	transducin-like enhancer of split 1 (E(sp1) homolog, Drosophila)	2.62	0.001484
C20orf108	chromosome 20 open reading frame 108	2.62	0.000031
PRKAR2B	protein kinase, cAMP-dependent, regulatory, type II, beta	2.59	0.000123
HDHD3	haloacid dehalogenase-like hydrolase domain containing 3	2.57	0.002685
PARVB	parvin, beta	2.57	0.00026
ADORA2B	adenosine A2b receptor	2.56	0.006139
THBS1	thrombospondin 1	2.54	0.010131
TPM1	tropomyosin 1 (alpha)	2.52	0.001261
C17orf99	chromosome 17 open reading frame 99	2.52	0.009234
SLAMF1	signaling lymphocytic activation molecule family member 1	2.51	0.038275
KLF9	Kruppel-like factor 9	2.5	0.0017
MPP1	membrane protein, palmitoylated 1, 55kDa	2.5	0.000033
ANKRD57	ankyrin repeat domain 57	2.49	0.001585
ALOX5AP	arachidonate 5-lipoxygenase-activating protein	2.49	0.002462
AMMECR1	Alport syndrome, mental retardation, midface hypoplasia and elliptocytosis chromosomal region gene 1	2.48	0.000003
G6PD	glucose-6-phosphate dehydrogenase	2.48	0.000878
EMP3	epithelial membrane protein 3	2.47	0.000081
C1orf150	chromosome 1 open reading frame 150	2.46	0.018192
ST6GALNAC1	ST6 (alpha-N-acetyl-neuraminyl-2,3-beta-galactosyl-1,3)-N-acetylgalactosaminide alpha-2,6-sialyltransferase 1	2.46	0.007024
ERRFI1	ERBB receptor feedback inhibitor 1	2.43	0.039253
CD46	CD46 molecule, complement regulatory protein	2.43	0.002322
S100A10	S100 calcium binding protein A10	2.41	0.000162
HIPK3	homeodomain interacting protein kinase 3	2.41	0.00097
TNIK	TRAF2 and NCK interacting kinase	2.4	0.000191
IL9R	interleukin 9 receptor	2.4	0.000143
ALDH6A1	aldehyde dehydrogenase 6 family, member A1	2.4	0.006546
CTSH	cathepsin H	2.4	0.018772
NTRK1	neurotrophic tyrosine kinase, receptor, type 1	2.39	0.026085
STON1	stonin 1	2.39	0.006653
NCOA2	nuclear receptor coactivator 2	2.39	0.000103

ALAD	aminolevulinate, delta-, dehydratase	2.38	0.017645
FREQ	frequenin homolog (Drosophila)	2.38	0.002354
ALDOC	aldolase C, fructose-bisphosphate	2.38	0.040266
MAN1A1	mannosidase, alpha, class 1A, member 1	2.35	0.000176
TXNIP	thioredoxin interacting protein	2.34	0.001908
CCNA1	cyclin A1	2.33	0.007367
C19orf59	chromosome 19 open reading frame 59	2.33	0.036549
GAS2L1	growth arrest-specific 2 like 1	2.33	0.000389
EPAS1	endothelial PAS domain protein 1	2.32	0.000629
LRP11	low density lipoprotein receptor-related protein 11	2.32	0.000364
PRKAB1	protein kinase, AMP-activated, beta 1 non-catalytic subunit	2.32	0.005656
ERMAP	erythroblast membrane-associated protein (Scianna blood group)	2.3	0.000591
REEP3	receptor accessory protein 3	2.29	0.001283
ATP8B1	ATPase, class I, type 8B, member 1	2.28	0.049081
PRKCSH	protein kinase C substrate 80K-H	2.28	0.000032
IGFBP2	insulin-like growth factor binding protein 2, 36kDa	2.27	0.002099
TPSAB1	tryptase alpha/beta 1	2.26	0.017475
VEGFA	vascular endothelial growth factor A	2.25	0.007244
GEM	GTP binding protein overexpressed in skeletal muscle	2.25	0.004995
H1F0	H1 histone family, member 0	2.25	0.002134
IL1RL1	interleukin 1 receptor-like 1	2.24	0.016679
UBXN10	UBX domain protein 10	2.22	0.001364
MTSS1	metastasis suppressor 1	2.22	0.000029
S100A6	S100 calcium binding protein A6	2.21	0.000172
EPCAM	epithelial cell adhesion molecule	2.21	0.009886
PPP1R15A	protein phosphatase 1, regulatory (inhibitor) subunit 15A	2.21	0.000168
ADAMTS3	ADAM metallopeptidase with thrombospondin type 1 motif, 3	2.2	0.00016
PIP5K1B	phosphatidylinositol-4-phosphate 5-kinase, type I, beta	2.2	0.000685
WBP2	WW domain binding protein 2	2.2	0.000066
SLC24A3	solute carrier family 24 (sodium/potassium/calcium exchanger), member 3	2.2	0.018555
FOS	v-fos FBJ murine osteosarcoma viral oncogene homolog	2.19	0.021642
SNAP23	synaptosomal-associated protein, 23kDa	2.19	0.004031
RARRES1	retinoic acid receptor responder (tazarotene induced) 1	2.18	0.010656
C13orf15	chromosome 13 open reading frame 15	2.18	0.009746
RTN2	reticulon 2	2.18	0.00099
CD37	CD37 molecule	2.18	0.001087
FYN	FYN oncogene related to SRC, FGR, YES	2.17	0.011288
ZNF192	zinc finger protein 192	2.17	0.00248
WNK3	WNK lysine deficient protein kinase 3	2.17	0.041102
ANKRD37	ankyrin repeat domain 37	2.16	0.001488
AKAP12	A kinase (PRKA) anchor protein 12	2.16	0.02138

PTGS1	prostaglandin-endoperoxide synthase 1 (prostaglandin G/H synthase and cyclooxygenase)	2.16	0.000362
SLC25A37	solute carrier family 25, member 37	2.15	0.014699
P2RX5	purinergic receptor P2X, ligand-gated ion channel, 5	2.15	0.008397
ATP6V0A1	ATPase, H+ transporting, lysosomal V0 subunit a1	2.15	0.000093
ABHD14B	abhydrolase domain containing 14B	2.14	0.000417
BHLHE40	basic helix-loop-helix family, member e40	2.14	0.001208
AGPAT1	1-acylglycerol-3-phosphate O-acyltransferase 1 (lysophosphatidic acid acyltransferase, alpha)	2.14	0.000011
SLC44A2	solute carrier family 44, member 2	2.14	0.002525
LCN2	lipocalin 2	2.13	0.006845
TPST2	tyrosylprotein sulfotransferase 2	2.13	0.000207
CAMK1	calcium/calmodulin-dependent protein kinase I	2.11	0.001968
SOCS1	suppressor of cytokine signaling 1	2.11	0.000003
MAZ	MYC-associated zinc finger protein (purine-binding transcription factor)	2.11	0.00722
APOBEC3C	apolipoprotein B mRNA editing enzyme, catalytic polypeptide-like 3C	2.1	0.000087
EREG	epiregulin	2.09	0.037605
PIR	pirin (iron-binding nuclear protein)	2.09	0.002241
TIMP1	TIMP metallopeptidase inhibitor 1	2.09	0.008648
CDC42EP4	CDC42 effector protein (Rho GTPase binding) 4	2.09	0.000373
FECH	ferrochelatase (protoporphyria)	2.09	0.001234
MYRIP	myosin VIIA and Rab interacting protein	2.08	0.021268
PKIG	protein kinase (cAMP-dependent, catalytic) inhibitor gamma	2.08	0.006428
LOC541471	hypothetical LOC541471	2.07	0.000435
STXBP6	syntaxin binding protein 6 (amisyn)	2.07	0.004146
NAPA	N-ethylmaleimide-sensitive factor attachment protein, alpha	2.06	0.000412
TMEM214	transmembrane protein 214	2.05	0.012853
CPA3	carboxypeptidase A3 (mast cell)	2.05	0.001252
GALC	galactosylceramidase	2.05	0.00011
RAB6B	RAB6B, member RAS oncogene family	2.04	0.019713
PDZD8	PDZ domain containing 8	2.04	0.003795
FRAT1	frequently rearranged in advanced T-cell lymphomas	2.04	0.002574
ENSG00000183700	NA	2.04	0.00281
ADAM10	ADAM metallopeptidase domain 10	2.04	0.0029
BSG	basigin (Ok blood group)	2.04	0.000181
ELL2	elongation factor, RNA polymerase II, 2	2.03	0.000372
MOSPD3	motile sperm domain containing 3	2.03	0.000089
IL10RA	interleukin 10 receptor, alpha	2.03	0.01488
FHDC1	FH2 domain containing 1	2.02	0.009671
LPAR5	lysophosphatidic acid receptor 5	2.02	0.002706
SLC4A2	solute carrier family 4, anion exchanger, member 2 (erythrocyte membrane protein band 3-like 1)	2.01	0.001793

TAL1	T-cell acute lymphocytic leukemia 1	2	0.00034

Genes down-regulated in EPP

Gene Symbol	Gene Name	Fold change	P value
SPINK2	serine peptidase inhibitor, Kazal type 2 (acrosin-trypsin inhibitor)	-6.73	0.000456
NOG	noggin	-5.06	0.001137
C1QTNF4	C1q and tumor necrosis factor related protein 4	-4.96	0.001387
PROM1	prominin 1	-4.82	0.010952
UBTD2	ubiquitin domain containing 2	-4.59	0.000104
CHRDL1	chordin-like 1	-4.58	0.000011
BAALC	brain and acute leukemia, cytoplasmic	-4.49	0.000148
CTSZ	cathepsin Z	-4.18	0.000173
RGL4	ral guanine nucleotide dissociation stimulator-like 4	-4.12	0.000677
PTPRCAP	protein tyrosine phosphatase, receptor type, C-associated protein	-4.04	0.001413
AIM1	absent in melanoma 1	-4.02	0.014656
TNFSF13B	tumor necrosis factor (ligand) superfamily, member 13b	-3.98	0.000784
SELL	selectin L	-3.97	0.000034
ARMCX2	armadillo repeat containing, X-linked 2	-3.97	0.000015
LY75	lymphocyte antigen 75	-3.94	0.000002
MYO5C	myosin VC	-3.89	0
HLF	hepatic leukemia factor	-3.88	0.002206
MT-ND6	mitochondrially encoded NADH dehydrogenase 6	-3.87	0.000014
MPO	myeloperoxidase	-3.84	0.024036
LOC284422	similar to HSPC323	-3.78	0.000569
SORL1	sortilin-related receptor, L(DLR class) A repeats-containing	-3.72	0.00478
TFEC	transcription factor EC	-3.63	0.000013
HGF	hepatocyte growth factor (hepapoietin A; scatter factor)	-3.59	0.000648
SCHIP1	schwannomin interacting protein 1	-3.58	0.000015
MIRHG2	microRNA host gene 2 (non-protein coding)	-3.52	0.000059
IFI44	interferon-induced protein 44	-3.48	0.004774
CTSG	cathepsin G	-3.48	0.002935
DUSP6	dual specificity phosphatase 6	-3.47	0.0018
TMEM200A	transmembrane protein 200A	-3.46	0.000652
ENSG00000167912	NA	-3.43	0.000122
CTHRC1	collagen triple helix repeat containing 1	-3.41	0.000054
CXorf21	chromosome X open reading frame 21	-3.39	0.000638
GLIPR1	GLI pathogenesis-related 1	-3.38	0.002403
C12orf5	chromosome 12 open reading frame 5	-3.37	0.004099
NPR3	natriuretic peptide receptor C/guanylate cyclase C (atrionatriuretic peptide receptor C)	-3.33	0.000049
CXCR7	chemokine (C-X-C motif) receptor 7	-3.31	0.000253
C5orf23	chromosome 5 open reading frame 23	-3.31	0.007896
KIAA1274	KIAA1274	-3.31	0.000006

PLEKHO1	pleckstrin homology domain containing, family O member 1	-3.3	0.000034
KBTBD11	kelch repeat and BTB (POZ) domain containing 11	-3.27	0.000051
SLC16A14	solute carrier family 16, member 14 (monocarboxylic acid transporter 14)	-3.22	0.000915
SLC22A16	solute carrier family 22 (organic cation/carnitine transporter), member 16	-3.18	0.000327
GOLGA9P	golgi autoantigen, golgin subfamily a, 9 pseudogene	-3.18	0.001387
TAF15	TAF15 RNA polymerase II, TATA box binding protein (TBP)-associated factor, 68kDa	-3.17	0.001031
MN1	meningioma (disrupted in balanced translocation) 1	-3.17	0.002195
NEURL1B	neuralized homolog 1B (Drosophila)	-3.15	0.000005
CD200	CD200 molecule	-3.14	0.000074
EPB41L3	erythrocyte membrane protein band 4.1-like 3	-3.1	0.011381
SUCNR1	succinate receptor 1	-3.08	0.000456
PRAM1	PML-RARA regulated adaptor molecule 1	-3.08	0.019985
ATP8B4	ATPase, class I, type 8B, member 4	-3.02	0.001027
GIMAP7	GTPase, IMAP family member 7	-2.97	0.002201
DNLZ	DNL-type zinc finger	-2.96	0.000696
TNFSF4	tumor necrosis factor (ligand) superfamily, member 4	-2.95	0.013789
LTB	lymphotoxin beta (TNF superfamily, member 3)	-2.94	0.000258
GZMA	granzyme A (granzyme 1, cytotoxic T-lymphocyte-associated serine esterase 3)	-2.93	0.001155
SH3BP4	SH3-domain binding protein 4	-2.92	0.000822
TRIM22	tripartite motif-containing 22	-2.92	0.001888
PXDN	peroxidasin homolog (Drosophila)	-2.9	0.000035
MSRB3	methionine sulfoxide reductase B3	-2.87	0.004449
FAM169A	family with sequence similarity 169, member A	-2.86	0.000835
TCTEX1D1	Tctex1 domain containing 1	-2.82	0.003789
SPNS3	spinster homolog 3 (Drosophila)	-2.81	0.010428
HOXA9	homeobox A9	-2.8	0.012864
IFITM1	interferon induced transmembrane protein 1 (9-27)	-2.8	0.034237
GBP1	guanylate binding protein 1, interferon-inducible, 67kDa	-2.79	0.017261
ARMCX1	armadillo repeat containing, X-linked 1	-2.77	0.005759
CALN1	calneuron 1	-2.76	0.000036
PION	pigeon homolog (Drosophila)	-2.74	0.000029
UCHL1	ubiquitin carboxyl-terminal esterase L1 (ubiquitin thiolesterase)	-2.73	0.004048
CRHBP	corticotropin releasing hormone binding protein	-2.73	0.000679
LMNB1	lamin B1	-2.73	0
BZRAP1	benzodiazapine receptor (peripheral) associated protein 1	-2.72	0.000001
RASGRP2	RAS guanyl releasing protein 2 (calcium and DAG-regulated)	-2.71	0.000106
GPR84	G protein-coupled receptor 84	-2.65	0.000016
LOC643332	similar to Nonsecretory ribonuclease precursor (Ribonuclease US) (Eosinophil-derived neurotoxin) (RNase UpI-2) (Ribonuclease 2) (RNase 2)	-2.65	0.000513

RASGRP1	RAS guanyl releasing protein 1 (calcium and DAG-regulated)	-2.65	0.001957
HOXA7	homeobox A7	-2.64	0.000106
TMEM71	transmembrane protein 71	-2.64	0.005164
LOC729680	hypothetical protein LOC729680	-2.64	0.001055
HSF5	heat shock transcription factor family member 5	-2.63	0.000349
ZNF439	zinc finger protein 439	-2.63	0.00486
TIMP2	TIMP metallopeptidase inhibitor 2	-2.62	0.034073
IL12RB2	interleukin 12 receptor, beta 2	-2.59	0.000866
FAM26F	family with sequence similarity 26, member F	-2.59	0.016239
HOXA5	homeobox A5	-2.59	0.004721
DUSP4	dual specificity phosphatase 4	-2.59	0.000923
CD93	CD93 molecule	-2.59	0.004464
LOXL1	lysyl oxidase-like 1	-2.58	0.000676
NLRC3	NLR family, CARD domain containing 3	-2.58	0.002137
CYYR1	cysteine/tyrosine-rich 1	-2.58	0.014357
ERG	v-ets erythroblastosis virus E26 oncogene homolog (avian)	-2.57	0.001374
C1orf59	chromosome 1 open reading frame 59	-2.54	0.000001
ANKRD36B	ankyrin repeat domain 36B	-2.54	0.008665
IGHM	immunoglobulin heavy constant mu	-2.54	0.001262
MDFIC	MyoD family inhibitor domain containing	-2.53	0.000534
NPDC1	neural proliferation, differentiation and control, 1	-2.53	0.001252
EMP1	epithelial membrane protein 1	-2.52	0.00111
C9orf43	chromosome 9 open reading frame 43	-2.5	0.000034
TMSB15A	thymosin beta 15a	-2.5	0.010245
C1orf228	chromosome 1 open reading frame 228	-2.49	0.001539
GPR124	G protein-coupled receptor 124	-2.49	0.001546
IL17D	interleukin 17D	-2.48	0.00218
CPNE2	copine II	-2.48	0.007568
ZNF492	zinc finger protein 492	-2.46	0.005513
GPX7	glutathione peroxidase 7	-2.44	0.004387
SPARC	secreted protein, acidic, cysteine-rich (osteonectin)	-2.44	0.0046
C12orf75	chromosome 12 open reading frame 75	-2.43	0.00526
DLK1	delta-like 1 homolog (Drosophila)	-2.4	0.00929
NKG7	natural killer cell group 7 sequence	-2.39	0.011124
RNASE3	ribonuclease, RNase A family, 3 (eosinophil cationic protein)	-2.38	0.000573
ZNF257	zinc finger protein 257	-2.38	0.000433
ZNF662	zinc finger protein 662	-2.37	0.000716
GAPT	GRB2-binding adaptor protein, transmembrane	-2.37	0.001412
RTP4	receptor (chemosensory) transporter protein 4	-2.36	0.000586
ZNF738	zinc finger protein 738	-2.35	0.000664
FNBP1	formin binding protein 1	-2.34	0.000009
IMPA2	inositol(myo)-1(or 4)-monophosphatase 2	-2.32	0.001674

CCDC58	coiled-coil domain containing 58	-2.31	0.006058
CHST13	carbohydrate (chondroitin 4) sulfotransferase 13	-2.31	0.004531
C9orf91	chromosome 9 open reading frame 91	-2.31	0.002074
MALAT1	metastasis associated lung adenocarcinoma transcript 1 (non-protein coding)	-2.3	0.00039
CACHD1	cache domain containing 1	-2.29	0.000312
ZBTB8A	zinc finger and BTB domain containing 8A	-2.28	0.008525
TRBV27	T cell receptor beta variable 27	-2.28	0.014474
AMICA1	adhesion molecule, interacts with CXADR antigen 1	-2.28	0.022934
RAB27A	RAB27A, member RAS oncogene family	-2.28	0.000178
MGC29506	hypothetical protein MGC29506	-2.27	0.0023
GPR183	G protein-coupled receptor 183	-2.27	0.020849
FAM111B	family with sequence similarity 111, member B	-2.26	0.000053
PRRT3	proline-rich transmembrane protein 3	-2.24	0.000155
ATP9A	ATPase, class II, type 9A	-2.22	0.007067
PHGDH	phosphoglycerate dehydrogenase	-2.21	0.001139
CASP1	caspase 1, apoptosis-related cysteine peptidase (interleukin 1, beta, convertase)	-2.21	0.043319
ANPEP	alanyl (membrane) aminopeptidase	-2.21	0.001708
BAT2D1	BAT2 domain containing 1	-2.2	0.002105
GALNT3	UDP-N-acetyl-alpha-D-galactosamine:polypeptide N-acetylgalactosaminyltransferase 3 (GalNAc-T3)	-2.2	0.000256
HERC5	hect domain and RLD 5	-2.2	0.003103
DSE	dermatan sulfate epimerase	-2.18	0.007097
CLEC5A	C-type lectin domain family 5, member A	-2.16	0.045442
KIAA1841	KIAA1841	-2.15	0.001256
PRAGMIN	homolog of rat pragma of Rnd2	-2.15	0.005921
GARNL4	GTPase activating Rap/RanGAP domain-like 4	-2.15	0.003128
FAM43A	family with sequence similarity 43, member A	-2.13	0.001233
RGS19	regulator of G-protein signaling 19	-2.13	0.002537
CERK	ceramide kinase	-2.12	0.000077
ASGR1	asialoglycoprotein receptor 1	-2.11	0.00022
BEX2	brain expressed X-linked 2	-2.09	0.017168
NUTF2	nuclear transport factor 2	-2.09	0.01493
ALCAM	activated leukocyte cell adhesion molecule	-2.08	0.015837
S100Z	S100 calcium binding protein Z	-2.08	0.000753
ZC3H12D	zinc finger CCCH-type containing 12D	-2.08	0.00906
SOX4	SRY (sex determining region Y)-box 4	-2.08	0.000171
ATL1	atlastin GTPase 1	-2.08	0.000343
SH3TC1	SH3 domain and tetratricopeptide repeats 1	-2.07	0.001603
NRIP3	nuclear receptor interacting protein 3	-2.07	0.001599
PSTPIP1	proline-serine-threonine phosphatase interacting protein 1	-2.07	0.002279
SNX10	sorting nexin 10	-2.06	0.000125
NSUN6	NOL1/NOP2/Sun domain family, member 6	-2.06	0.000316

DNAJC17	DnaJ (Hsp40) homolog, subfamily C, member 17	-2.06	0.000093
SIDT1	SID1 transmembrane family, member 1	-2.05	0.000065
PABPC4L	poly(A) binding protein, cytoplasmic 4-like	-2.05	0.000015
IFI16	interferon, gamma-inducible protein 16	-2.04	0.014353
P2RY8	purinergic receptor P2Y, G-protein coupled, 8	-2.04	0.003957
STYK1	serine/threonine/tyrosine kinase 1	-2.04	0.010581
ZNF573	zinc finger protein 573	-2.04	0.047831
BAX	BCL2-associated X protein	-2.04	0.002302
RHOBTB1	Rho-related BTB domain containing 1	-2.03	0.021154
CD34	CD34 molecule	-2.02	0.013742
RSPH10B2	radial spoke head 10 homolog B2 (Chlamydomonas)	-2.02	0.000832
BEX5	brain expressed, X-linked 5	-2.02	0.001639
TMEM163	transmembrane protein 163	-2.01	0.017745
FSTL1	follistatin-like 1	-2.01	0.002255
SLC22A4	solute carrier family 22 (organic cation/ergothioneine transporter), member 4	-2.01	0.001119
SGK3	serum/glucocorticoid regulated kinase family, member 3	-2.01	0.002213
CORO1A	coronin, actin binding protein, 1A	-2.01	0.000244
TARDBP	TAR DNA binding protein	-2	0.000083

Genes up-regulated in MPP

Gene Symbol	Gene Name	Fold change	P value
S100A8	S100 calcium binding protein A8	6.91	0.000007
PRTN3	proteinase 3	5.78	0.000368
ELANE	elastase, neutrophil expressed	5.41	0.006886
CLC	Charcot-Leyden crystal protein	4.67	0.018955
AZU1	azurocidin 1	4.57	0.000465
FOS	v-fos FBJ murine osteosarcoma viral oncogene homolog	3.94	0.003005
CD24	CD24 molecule	3.91	0.000881
S100A9	S100 calcium binding protein A9	3.88	0.001064
ALOX5	arachidonate 5-lipoxygenase	3.85	0.011731
VCAN	versican	3.77	0.001689
SLPI	secretory leukocyte peptidase inhibitor	3.71	0.001067
HBB	hemoglobin, beta	3.68	0.005643
C19orf59	chromosome 19 open reading frame 59	3.58	0.000217
PRG2	proteoglycan 2, bone marrow (natural killer cell activator, eosinophil granule major basic protein)	3.54	0.015258
MNDA	myeloid cell nuclear differentiation antigen	3.49	0.000992
MS4A6A	membrane-spanning 4-domains, subfamily A, member 6A	3.43	0.003291
SERPINB10	serpin peptidase inhibitor, clade B (ovalbumin), member 10	3.43	0.000376
CSTA	cystatin A (stefin A)	3.36	0.000171
CST7	cystatin F (leukocystatin)	3.35	0.00017
STON1	stonin 1	3.34	0.000823
HP	haptoglobin	3.34	0.000556
C5orf20	chromosome 5 open reading frame 20	3.3	0.000751
MS4A3	membrane-spanning 4-domains, subfamily A, member 3 (hematopoietic cell-specific)	3.2	0.007733
CFD	complement factor D (adipsin)	3.07	0.004227
S100P	S100 calcium binding protein P	3.03	0.006748
NCF2	neutrophil cytosolic factor 2	3	0.002454
RETN	resistin	2.94	0.001154
HCK	hemopoietic cell kinase	2.89	0.000786
CD1D	CD1d molecule	2.81	0.005186
RAB20	RAB20, member RAS oncogene family	2.8	0.000007
PIWIL4	piwi-like 4 (Drosophila)	2.79	0.002774
ENPP2	ectonucleotide pyrophosphatase/phosphodiesterase 2	2.72	0.002343
P2RY2	purinergic receptor P2Y, G-protein coupled, 2	2.69	0.002087
LOC283663	hypothetical LOC283663	2.69	0.007697
LYZ	lysozyme (renal amyloidosis)	2.62	0.002217
ADAMDEC1	ADAM-like, decysin 1	2.61	0.011886
CNRIP1	cannabinoid receptor interacting protein 1	2.6	0.02062
ANXA3	annexin A3	2.6	0.000589
PARP8	poly (ADP-ribose) polymerase family, member 8	2.54	0.000548

HAL	histidine ammonia-lyase	2.53	0.000282
LIN7A	lin-7 homolog A (C. elegans)	2.5	0.00381
EGID-79948	plasticity-related gene 2	2.5	0.000003
CTSG	cathepsin G	2.47	0.000223
ELOVL3	elongation of very long chain fatty acids (FEN1/Elo2, SUR4/Elo3, yeast)-like 3	2.46	0.009838
S100A12	S100 calcium binding protein A12	2.44	0.01207
SERPINB2	serpin peptidase inhibitor, clade B (ovalbumin), member 2	2.44	0.009607
P2RY13	purinergic receptor P2Y, G-protein coupled, 13	2.43	0.000658
CEACAM8	carcinoembryonic antigen-related cell adhesion molecule 8	2.43	0.012363
PLBD1	phospholipase B domain containing 1	2.39	0.029536
OLR1	oxidized low density lipoprotein (lectin-like) receptor 1	2.33	0.04844
CLEC12A	C-type lectin domain family 12, member A	2.33	0.003719
SLC22A15	solute carrier family 22, member 15	2.32	0.000013
CD14	CD14 molecule	2.31	0.003414
DYSF	dysferlin, limb girdle muscular dystrophy 2B (autosomal recessive)	2.28	0.001532
TGFBI	transforming growth factor, beta-induced, 68kDa	2.28	0.009135
ASGR2	asialoglycoprotein receptor 2	2.27	0.000117
NAPSB	napsin B aspartic peptidase pseudogene	2.27	0.000762
MPEG1	macrophage expressed 1	2.26	0.005274
LRG1	leucine-rich alpha-2-glycoprotein 1	2.24	0.004884
FCER1G	Fc fragment of IgE, high affinity I, receptor for; gamma polypeptide	2.22	0.002326
CD36	CD36 molecule (thrombospondin receptor)	2.22	0.000078
BEX1	brain expressed, X-linked 1	2.22	0.044141
IPCEF1	interaction protein for cytohesin exchange factors 1	2.21	0.007888
FZD2	frizzled homolog 2 (Drosophila)	2.21	0.021064
TCN1	transcobalamin I (vitamin B12 binding protein, R binder family)	2.2	0.049048
CEACAM6	carcinoembryonic antigen-related cell adhesion molecule 6 (non-specific cross reacting antigen)	2.2	0.022493
C12orf59	chromosome 12 open reading frame 59	2.18	0.004136
RNASE2	ribonuclease, RNase A family, 2 (liver, eosinophil-derived neurotoxin)	2.16	0.000946
FGR	Gardner-Rasheed feline sarcoma viral (v-fgr) oncogene homolog	2.13	0.000647
CLU	clusterin	2.13	0.005949
GPR160	G protein-coupled receptor 160	2.12	0.002806
KCNH2	potassium voltage-gated channel, subfamily H (eag-related), member 2	2.12	0.008066
ALOX5AP	arachidonate 5-lipoxygenase-activating protein	2.12	0.002995
LY86	lymphocyte antigen 86	2.11	0.003033
CEBPD	CCAAT/enhancer binding protein (C/EBP), delta	2.11	0.002477
TYROBP	TYRO protein tyrosine kinase binding protein	2.1	0.00055
ACPP	acid phosphatase, prostate	2.09	0.000879

ALAS1	aminolevulinate, delta-, synthase 1	2.06	0.000194
RNASE6	ribonuclease, RNase A family, k6	2.06	0.000505
FCN1	ficolin (collagen/fibrinogen domain containing) 1	2.02	0.010237
SEPP1	selenoprotein P, plasma, 1	2.01	0.000268
SLC47A1	solute carrier family 47, member 1	2.01	0.00274
PCOLCE2	procollagen C-endopeptidase enhancer 2	2	0.001528
ALOX12	arachidonate 12-lipoxygenase	2	0.004849
CILP2	cartilage intermediate layer protein 2	2	0.001953

Genes down-regulated in MPP

Gene Symbol	Gene Name	Fold change	P value
DLK1	delta-like 1 homolog (Drosophila)	-3.96	0.000169
TMEM200A	transmembrane protein 200A	-3.55	0.000282
ARMCX2	armadillo repeat containing, X-linked 2	-3.52	0.000017
TMSB15A	thymosin beta 15a	-3.37	0.00702
HLF	hepatic leukemia factor	-3.3	0.002439
BEX2	brain expressed X-linked 2	-3.26	0.000961
CRHBP	corticotropin releasing hormone binding protein	-3.25	0.000809
MMRN1	multimerin 1	-3.09	0.000266
SLC16A14	solute carrier family 16, member 14 (monocarboxylic acid transporter 14)	-3.07	0.001477
SPINK2	serine peptidase inhibitor, Kazal type 2 (acrosin-trypsin inhibitor)	-3	0.000094
CNN3	calponin 3, acidic	-2.97	0.000004
IFI44	interferon-induced protein 44	-2.86	0.000719
GZMA	granzyme A (granzyme 1, cytotoxic T-lymphocyte-associated serine esterase 3)	-2.84	0.001536
AKR1C3	aldo-keto reductase family 1, member C3 (3-alpha hydroxysteroid dehydrogenase, type II)	-2.79	0.010452
HEMGN	hemogen	-2.74	0.000021
GBP1	guanylate binding protein 1, interferon-inducible, 67kDa	-2.69	0.017717
C5orf23	chromosome 5 open reading frame 23	-2.53	0.000016
RAI14	retinoic acid induced 14	-2.53	0.000382
HTR1F	5-hydroxytryptamine (serotonin) receptor 1F	-2.47	0.000132
TGFB1I1	transforming growth factor beta 1 induced transcript 1	-2.46	0.004316
TNFSF4	tumor necrosis factor (ligand) superfamily, member 4	-2.43	0.000365
CTHRC1	collagen triple helix repeat containing 1	-2.43	0.000037
ABCB1	ATP-binding cassette, sub-family B (MDR/TAP), member 1	-2.41	0.000056
CD34	CD34 molecule	-2.38	0.000183
NAP1L3	nucleosome assembly protein 1-like 3	-2.36	0.001473
IL12RB2	interleukin 12 receptor, beta 2	-2.34	0.000333
CHRDL1	chordin-like 1	-2.34	0.00185
GNAI1	guanine nucleotide binding protein (G protein), alpha inhibiting activity polypeptide 1	-2.27	0.000131
KIAA0125	KIAA0125	-2.27	0.000239
NPR3	natriuretic peptide receptor C/guanylate cyclase C (atrionatriuretic peptide receptor C)	-2.24	0.000253
SH3BP5	SH3-domain binding protein 5 (BTK-associated)	-2.19	0.00922
GUCY1B3	guanylate cyclase 1, soluble, beta 3	-2.18	0.000195
PLCB4	phospholipase C, beta 4	-2.17	0.000004
FAM111B	family with sequence similarity 111, member B	-2.16	0.011623
RASGRP1	RAS guanyl releasing protein 1 (calcium and DAG-regulated)	-2.16	0.001808
CALN1	calneuron 1	-2.1	0.000011

SMAGP	small trans-membrane and glycosylated protein	-2.1	0.000038
EMP1	epithelial membrane protein 1	-2.1	0.006898
TMEFF1	transmembrane protein with EGF-like and two follistatin-like domains 1	-2.09	0.000108
ASAP2	ArfGAP with SH3 domain, ankyrin repeat and PH domain 2	-2.05	0.005281
CXCR7	chemokine (C-X-C motif) receptor 7	-2.02	0.003424

Supplemental Table 2. Distribution of the number of CAGE promoters per known gene-transcript.

# Promoters	# Known genes-transcripts HSPC	# Known genes-transcripts EPP	# Known genes-transcripts MPP
1	8908	9011	8844
2	924	935	893
3	86	94	83
4	11	12	10
5	2	2	3
6	1	1	0
Total	9932	10055	9833

Supplemental Table 3. List of HSPC-, EPP- and MPP- specific transcription factors

HSPC	EPP	MPP
DNMT3A	CREB3L3	ARID4A
HOXA7	EGR1	ASH1L
MYCN	FBXO7	BAZ2B
NAP1L3	FHL2	C21orf66
SLA2	GATA1	CNOT6
	GFI1B	CREB5
	HES6	DENND4A
	KLF1	FOS
	KLF3	GTF2A1
	LDB1	ID3
	MXI1	JARID2
	NFE2	KLF4
	SEC14L2	LRRFIP1
	STAT5A	MEF2A
	TAL1	MLL3
	TEAD1	MLLT10
	TSC22D1	MNDA
	TSC22D3	MXD4
	ZBTB16	NCOA4
	ZFPM1	NFIA
		NFIL3
		NR2C1
		REL
		RREB1
		SFRS14
		SHPRH
		SREBF1
		STAT6
		TBC1D22A
		ZBTB20
		ZBTB7B
		ZNF587
		ZNF70

Supplemental Table 4. ChIP-defined promoters in multipotent and lineage-restricted hematopoietic progenitors.

Cell type	# Promoters	Average length (kb)	% H3K27ac$^+$ Promoters	% Transcribed Promoters
**HSPC**	19449	2.6	55.6	44.8
**EPP**	12671	3.2	67.8	65.1
**MPP**	14078	2.7	54.6	58.2

Supplemental Table 5. ChIP-defined enhancers in multipotent and lineage-restricted hematopoietic progenitors.

Cell type	# Enhancers	Average length (kb)	% H3K27ac^{+} Enhancers	% Transcribed Enhancers
**HSPC**	65031	2.7	21.3	0.4
**EPP**	49421	3.5	25.1	0.6
**MPP**	66909	3.2	11.1	0.4

Supplemental Table 6. Super-enhancers in multipotent and lineage-restricted hematopoietic progenitors.

Cell type	# Super-enhancers	Average length (kb)	% Transcribed Super-enhancers
**HSPC**	755	45.2	80.8
**EPP**	513	45.6	71.5
**MPP**	600	18.6	55.3

SUPPLEMENTAL MATERIALS AND METHODS

Purification of HSPC, EPP and MPP

We isolated mononuclear cells by Ficoll-Hypaque (Lymphoprep; Sentinel Diagnostics) gradient separation and purified $CD34^+$ cells by immunomagnetic sorting (EasySep Human CD34 Positive Selection kit, StemCell Technologies Inc.).

Isolation of HSPC

We seeded $CD34^+$ cells at 0.5-1x10^6 cells/ml and cultured them for 36h in IMDM medium (Lonza) containing 20% fetal bovine serum (FBS) (Hyclone) and supplemented with 100 ng/ml human stem cell factor (hSCF), 100 ng/ml human Flt3-ligand (hFlt3-l), 20 ng/ml human trombopoietin (hTPO) and 20 ng/ml human Interleukin-6 (hIL-6) (all PeproTech). After 36h, we labeled the cells with fluorescein isothiocyanate (FITC)-conjugated anti-CD34, phycoerythrin (PE)-conjugated anti-CD133 and tri-color (TC) anti-CD38 antibodies. We sorted $CD34^+CD133^+$ multipotent progenitors using a MoFlo cell sorter (Beckman Coulter).

Isolation of EPP

We cultured $CD34^+$ cells for 5 days as described by Roselli et al. [1]. We seeded the cells at 10^5 cells/ml in StemSpan medium (Stem Cell Technologies) containing 20% FBS (Hyclone) and supplemented with 50 ng/ml hSCF (Peprotech), 1 U/ml human erythropoietin (EPO) (Janssen), 1 ng/ml hIL-3 (Peprotech), 10^{-6} M dexamethasone (Sigma), and 10^{-6} M ß-estradiol (Sigma). At day 5, we labeled the cells with FITC-conjugated anti-CD36 antibody and sorted $CD36^+$ erythroid progenitors using a MoFlo cell sorter (Beckman Coulter). We also stained $CD36^+$ cells with PE-conjugated anti-CD34, Allophycocyanin (APC)-conjugated anti-glycophorin A and Peridinin chlorophyll- conjugated (PerCP) anti-CD71 antibodies.

Isolation of MPP

We cultured $CD34^+$ cells at 10^5 cells/ml in IMDM medium (Lonza) containing 10% FBS (Hyclone) and supplemented with 100 ng/ml hSCF (Peprotech), 20 ng/ml hIL-3 (Peprotech), 100 ng/ml G-CSF (ITALFARMACO SpA). At day 5, we stained the cells with FITC-conjugated anti-CD34 and PE-conjugated anti-CD13 antibodies and purified $CD13^+CD34^-$ MPP using a MoFlo cell sorter (Beckman Coulter). We also labeled MPP with APC-conjugated anti-CD33 or APC-conjugated anti-CD11b antibodies.

We performed analyses by fluorescence-activated cell-sorter (FACS) using FACSCanto flow cytometer (BD Biosciences). Antibodies used for FACS analysis are listed below:

Antibody	Catalog #	Company
GpA-PE	R7078	Dako
GpA-APC	551336	BD Pharmingen
CD11b-APC	553312	BD Pharmingen
CD11b-FITC	553310	BD Pharmingen
CD11b-pe	553311	BD Pharmingen
CD33- APC	551378	BD Pharmingen
CD13-PE	MHCD1304	CALTAG
CD34-FITC	345801	BD Pharmingen
CD34-PE	345802	BD Pharmingen
CD36-FITC	555454	BD Pharmingen
CD38-APC	555462	BD Pharmingen
CD71-PE	IM2001U	Beckman Coulter
CD71 PerCP	551374	BD Pharmingen
CD133/2(293C3)-PE	130-090-853	Miltenyi Biotech
CD14-FITC	555393	BD Pharmingen
CD117-PE	332785	BD Biosciences

CFU assay
We plated multipotent and lineage–committed progenitors cells at $1x10^3$ cells/ml in methylcellulose medium (GFH4434, Stem Cell Technologies) under conditions supporting both erythroid and myelomonocytic differentiation. We scored BFU-E, CFU-GM and CFU-GEMM colonies after 14 days.

Erythroid and myeloid liquid culture
We performed *in vitro* erythroid differentiation as described by Roselli et al. [1]. For myeloid differentiation, we cultured $CD34^+$ cells for 7 days as described above (see "Isolation of MPP"). At day 7, we maintained the cells for 7 days in IMDM medium (Lonza) containing 10% FBS (Hyclone) and supplemented with 100 ng/ml G-CSF (ITALFARMACO SpA).

CAGE
Library preparation, sequencing and mapping
We extracted RNA from multipotent and lineage-committed progenitors using RNeasy Plus Mini kit (QIAGEN). DNAFORM Inc. at RIKEN Omics Science Center (Japan) performed DeepCAGE library preparation. Briefly, the cDNA synthesis was performed with 5 µg of total RNA, the random (N15) reverse-transcription primers and PrimeScript Reverse Transcriptase (TAKARA). Capped RNA was biotinylated and treated with RnaseOne. Then, hybrid cDNA with biotinylated Capped RNA was selected with cap-trapper method [2]. cDNA was released from streptavidin beads. A sample-specific linker, containing a recognition site for the sample-specific barcode sequence (3 bp) and the type III restriction-modification enzyme EcoP15I, was ligated to the single-strand cDNA. After ligation, the 2nd strand synthesis was performed and the resulting double-stranded cDNA was cleaved with EcoP15I. After, a second linker was ligated to the CAGE tag. The CAGE tags were separated from unmodified DNA with streptavidin beads. Then, the DNA fragments were PCR-amplified by using linker-specific primers. Samples were sequenced using the Illumina GAIIx sequencer, with an average read length of 36 bases. Each library was sequenced in one lane of a single strand 38 bp Illumina Run. 13 to 15 million reads were obtained per lane.
DNAFORM performed CAGE data analysis. Briefly, tags were extracted and mapped to human genome version hg19 (NCBI build 37), with a minimum match length of 21 bases and a maximum of one error; tags mapping the human ribosomal DNA sequence (representing 0.9-2.5% of the total CAGE tags) were eliminated. For CAGE tags mapping to multiple genome locations, a weighting strategy, based on the number of CAGE tags within a 200bp neighborhood around each candidate mapping location, was applied. Equal weights were used if no unique tags were found within the 200 bp region for all candidate mapping locations[3]. The average mapping rate was 40%. We obtained 5,128,106, 6,144,954and 5,210,354 mapped CAGE tags in HSPC, EPP and MPP, respectively. These numbers met the criteria used by the FANTOM5 consortium, where CAGE libraries were analyzed if containing at least 500,000 successfully aligned reads [4,5].

Sample	Total CAGE Tags*	Total Mapped CAGE Tags	Uniquely Mapped CAGE Tags	Multi-mapped CAGE Tags
HSPC	12,910,575	5,128,106	4,331,577	796,529
EPP	14,980,057	6,144,954	4,700,060	1,444,894
MPP	13,744,962	5,210,354	4,429,486	780,868

*After rDNA removal

Promoter construction
We defined level-1 promoters ("transcription start sites") by summing the weighted number of CAGE tags at each genome position. 567254, 560724 and 651829 TSSs were identified in HSPC, EPP and MPP, respectively. Then we clustered level-1 promoters into level-2 promoters ("CAGE promoters") if they were within 20 bp of each other on the same

chromosomal strand. Level-2 clustering defined >13,000 promoters of similar average length (160 ±101 bp) in each cell type (13,852 in HSPC, 13,609 in MPP and 14,041 in EPP). We calculated the expression level for each level-1 and level-2 promoter by dividing the number of CAGE tags of each promoter in each experimental condition by the total number of mapped CAGE tags in that condition, and multiplying by 1,000,000 (tags-per-million, TPM). The expression of each level-2 promoter of at least 10 TPM in at least one experimental condition was imposed.

Promoter genomic annotation
Using a custom R script, we annotated level-2 promoters using RefSeq genes, ENSEMBL ncRNA, ncRNA included in publicly available data sets [6 7 8], Vertebrate Genome Annotation (Vega) pseudogenes [9], Yale Gerstein Group pseudogenes [10] and GENCODE (release 14). For each dataset, the script performed the annotation considering the smallest distance to the CAGE-defined promoter on the same chromosome strand. We defined the distance as follows: 1. If the 3' end of the promoter was upstream of the 5' end of the annotation, then we used the distance between the 3' end of the promoter and the 5' end of the annotation. 2. If the 5' end of the promoter was downstream of the 5' end of the annotation, then we used the distance between the 5' of the annotation and the 5' end of the promoter. 3. If the promoter overlapped the 5' end of the annotation, we considered the distance to be zero. If this distance was less than 400 bp, we associated the promoters to the gene or transcript.

Statistical analyses
To compare CAGE and microarray expression levels, we calculated the Pearson correlation coefficients of CAGE log2 tpm counts and log2 expression values for the CAGE promoters and the Affymetrix probe sets annotated to the same gene. For genes associated to more than 1 CAGE promoter, we calculated the average expression level of all the CAGE promoters assigned to the gene.
We used the chi-squared test and a threshold p-value <0.001 to analyze differentially expressed CAGE level 2 promoters [11].

qRT-PCR
RNA was reverse-transcribed using Superscript II as per the manufacturer’s instructions (Invitrogen). Quantitative reverse-transcription PCR (qRT-PCR) was performed using SYBR green (Applied Biosystems).

ChIP-seq
ChIP assay
We prepared chromatin from EPP and MPP after cross-linking for 10’ at RT with 1% formaldehyde-containing medium, using *tru*ChIP™ High Cell Chromatin Shearing Kit with SDS Shearing Buffer (Covaris). We sonicated nuclear extracts to obtain DNA fragments averaging 200 bp in length and immunoprecipitated the equivalent of 10^7 cells overnight with 10 µg of rabbit antibodies against H3K4me1 (ab8895, Abcam), H3K4me3 (ab8580, Abcam), and H3K27ac (ab4729, Abcam), as previously described [12,13]. We used real-time SYBR Green PCR to validate genomic regions enriched in H3K4me1, H3K4me3 and H3K27ac in 3 biological replicates. Four genomic regions were used as positive (HBG promoter, GATA1 binding site, CSF3R promoter) and negative (DEFB122) controls, as shown below.

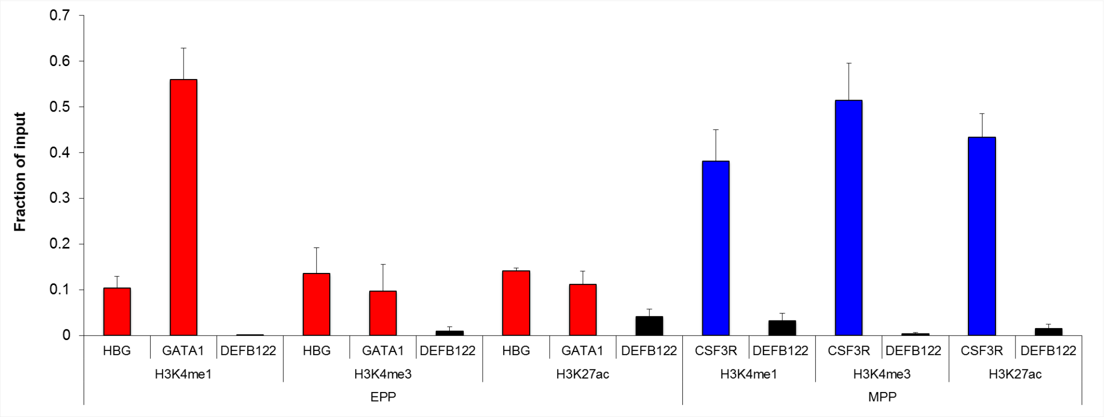

ChIP Seq library preparation and sequencing

We prepared Illumina libraries, for EPP and MPP, from 10 ng of immunoprecipitated DNA (IP) and control DNA (INPUT: nuclear extracts sonicated but non-immunoprecipitated) following the Illumina ChIP-seq DNA sample preparation kit. We checked the libraries by capillary electrophoresis by Agilent Bioanalyzer 2100 with the High sensitivity DNA assay and quantified them with Quant-iT™ PicoGreen ® dsDNA Kits (Invitrogen) by Nanodrop Fluorometer. We sequenced each library in one lane of a single strand 50 bp Illumina Run.

Bioinformatic ChIP-seq data Analysis

We mapped raw reads against the human reference genome (build hg19) using Bowtie [14] allowing up to 2 or 3 mismatches. We then processed each BAM file by using SAMtools [15], and converted each into a bed file using BEDTools [16]. Then we checked the quality of each sequenced sample using FastQC[17] cross-correlation analysis implemented in spp R package [18] and ENCODE quality metrics[19]. We performed ChIP-seq peak calling using SICER default parameters [20] and using each INPUT data to model the background noise. We downloaded HSPC raw H3K4me3, H3K4me1 and H3K27ac ChIP-seq data from the NIH Roadmap Epigenomics Mapping Consortium database (GSM772950, GSM773041, GSM773043, GSM772894, GSM772885 and GSM772870) and analyzed them as described above for EPP and MPP. ChIP-seq data used in this study are listed below:

Sample	Mapped reads	NRF	FRiP	NSC	RSC	Enriched regions
HSPC_H3K4me3*	31,131,313	0.78	0.82	1.35	1.21	48,022
HSPC_H3K4me1*	31,276,729	0.85	0.74	1.12	1.25	90,544
HSCP_H3K27ac*	31,602,356	0.40	0.40	1.15	4.12	26,932
HSCP_input*	39,436,811	0.97	-	-	-	-
EPP_H3K4me3	28,128,391	0.75	0.85	1.61	1.06	27,172
EPP_H3K4me1	26,185,846	0.94	0.32	1.21	1.13	68,418
EPP_H3K27ac	25,763,530	0.72	0.07	1.13	3.44	28,025
EPP_input	20,272,745	0.98	-	-	-	-
MPP_H3K4me3	28,732,471	0.43	0.81	1.77	1.13	30,346
MPP_H3K4me1	27,091,353	0.71	0.19	1.14	1.23	86,741
MPP_H3K27ac	37,720,127	0.30	0.12	2.28	13.59	24,584
MPP_input	23,370,210	0.54	-	-	-	-

NRF: Non-Redundant Fraction of mapped reads; FRiP: Fraction of Reads inside Peaks; NSC: normalized strand coefficient; RSC: relative strand correlation. *data from GSE17312.

Identification of cis-regulatory elements

We developed a custom R-workflow to identify promoters and enhancers. The pipeline analyzes the histone modification islands generated by SICER and includes three steps. In the first step, the R script invokes BEDtools [16] to detect regions where H3K4me1 overlap or do

not overlap with H3K4me3. Regions marked exclusively with H3K4me3 or H3K4me1 were classified as putative promoters and enhancers, respectively. In the second step, H3K4me3^{+} H3K4me1^{-} and H3K4me3^{-}H3K4me1^{+} regions are classified as putative promoters and enhancers, respectively. In this step, the R script first normalizes the tag counts of H3K4me3 and H3K4me1 using the sequencing depths of both libraries and then calculates the log-ratios between H3K4me3 and H3K4me1 tag counts for H3K4me3^{+}H3K4me1^{+} regions. If the H3K4me3/H3K4me1 ratio is greater than 0, the region is defined as putative promoter, otherwise as putative enhancer. In addition, we defined putative enhancers as H3K4me3^{-} /me1^{high} and H3K4me3^{low}/me1^{high} regions 2kb far from TSSs of annotated CAGE promoters and RefSeq genes. Finally, we intersected putative promoters and enhancers with H3K27ac^{+} regions to identify active chromatin regions.

Definition of super-enhancers

H3K27ac peaks were used as constituent enhancers for super-enhancers identification. Enhancers were stitched and super-enhancers were defined using ROSE code[21], as already described [22]. Briefly, the algorithm stitches enhancers together if they lie within a certain distance and ranks the enhancers by their input-subtracted signal of H3K27ac. It then separates super-enhancers from typical enhancers by identifying an inflection point of H3K27ac signal versus enhancers rank. ROSE was run with stitching distance of 12,500 bp. In addition, all the enhancers wholly contained in a window ± 2,500 bp around an annotated transcriptional start site (RefSeq hg19) were excluded from stitching, allowing for a total 5,000 bp promoter exclusion zone. Super-enhancers were then assigned to the RefSeq gene whose TSS was the nearest to the center of the stitched enhancers.

Retroviral scanning

Sequencing and mapping of retroviral integration sites

Retroviral integration sites were cloned by linker-mediated PCR (LM-PCR) adapted to the GS-FLX Genome Sequencer (Roche/454 Life Sciences, Branford, CT) pyrosequencing platform, as previously described[23]. Briefly, genomic DNA was extracted and digested with *Mse*I and a second enzyme *PstI* to prevent amplification of internal 3' LTR fragments. An *Mse*I double-stranded linker was then ligated and LM-PCR performed with nested primers specific for the linker and the 3' LTR, containing a bead-capture tag and a sequencing tag.Raw sequence reads were processed and mapped onto the human genome by an automated bioinformatics pipeline that eliminates viral and linker sequences, as previously described[23]. Unique sequences 20-bp or longer were mapped onto the human genome (UCSC Human Genome Project Working Draft, hg19) using Blat sequence alignment tool, requiring univocal match and a 95% identity over the entire sequence length.

Sample	Total raw reads	Unique sequences >20bp	Unique integration sites
HSPC	244,879	73,180	32,574
EPP	409,881	81,955	27,546
MPP	173,182	70,005	36,358

Statistical definition of retroviral integration clusters in HSPC, EPP and MPP.

We plotted the percentage distribution of the distances (log scale on the x axis) between any insertion site and the second consecutive integration in the MLV datasets (red lines), together with the same number of control random sites (black lines). Control sites were re-sampled 1,000 times from the entire collection of 11,655,601 random sites. Dashed vertical lines identify the threshold in the normal site distribution containing 1% of the sites, representing the false discovery rate (FDR) for cluster definition. A bimodal distribution was observed for MLV, identifying two populations, one (left part) of highly clustered integration, and the other (right part) almost coinciding with the random curve. We chose the distance between three consecutive integrations and a 1% FDR as a threshold for cluster definition (12,598,

14921 and 10,169bp for HSPC, EPP and MPP, respectively) as the best trade-off between low background noise and good discrimination of the two MLV populations (as described in[23]).

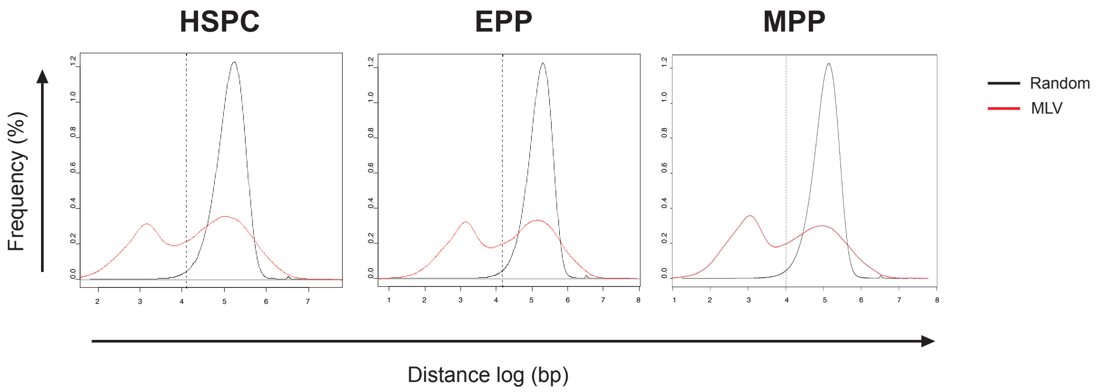
HSPC
EPP
MPP
Random
MLV
Frequency (%)
Distance log (bp)

Luciferase reporter assay

We amplified by PCR eight EPP- and MPP-specific MLV-targeted putative enhancers and cloned them upstream of a minimal promoter-Firefly reporter cassette in the pGL4.23 vector (Promega). We used two negative controls of different size (1.6 or 2.4 kb) containing a genomic region lacking any MLV cluster or ChIP-defined enhancer. pGL4.13 vector (Promega), containing a SV40 promoter-Firefly luciferase reporter cassette, served as positive control. We nucleofected 10^6 EPP or MPP with 2 µg of test plasmid and 10 ng of pGL4.73 vector, a reporter plasmid expressing Renilla luciferase driven by SV40 promoter (Promega), using the 4D-Nucleofector™ System (Lonza, program EO-100). 18 hours after transfection, we analyzed cell extracts using the Dual Luciferase assay kit according to manufacturer's instructions (Promega). We normalized Firefly luciferase activity to Renilla luciferase signal. Then, we calculated the fold changes between normalized Firefly luciferase activities of tested and control plasmids. Finally, we calculated the log2 of the ratio between EPP and MPP fold induction for each enhancer. Genomic coordinates of enhancers tested in luciferase assay are listed below:

**Name**	**Chr**	**Start**	**End**
#1	chr16	88577364	88578875
#2	chr22	31979743	31982505
#3	chr4	79652836	79655550
#4	chr4	55436390	55438978
#5	chr4	1929487	1931487
#6	chr5	148808828	148811016
#7	chr7	1231965	1234105
#8	chr18	21599148	21601261
#Neg Ctrl 1	chr8	49821736	49823355
#Neg Ctrl 2	chr8	49821738	49824134

REFERENCES

1 Roselli, E. A. *et al.* Correction of beta-thalassemia major by gene transfer in haematopoietic progenitors of pediatric patients. *EMBO Mol Med* **2**, 315-328, doi:10.1002/emmm.201000083 (2010).

2 Carninci, P. *et al.* High efficiency selection of full-length cDNA by improved biotinylated cap trapper. *DNA Res* **4**, 61-66 (1997).

3 Faulkner, G. J. *et al.* A rescue strategy for multimapping short sequence tags refines surveys of transcriptional activity by CAGE. *Genomics* **91**, 281-288, doi:10.1016/j.ygeno.2007.11.003 (2008).

4 Forrest, A. R. *et al.* A promoter-level mammalian expression atlas. *Nature* **507**, 462-470, doi:10.1038/nature13182 (2014).

5 Arner, E. *et al.* Gene regulation. Transcribed enhancers lead waves of coordinated transcription in transitioning mammalian cells. *Science (New York, N.Y* **347**, 1010-1014, doi:10.1126/science.1259418 (2015).

6 Khalil, A. M. *et al.* Many human large intergenic noncoding RNAs associate with chromatin-modifying complexes and affect gene expression. *Proc. Natl. Acad. Sci. USA* **106**, 11667-11672, doi:10.1073/pnas.0904715106 (2009).

7 Cabili, M. N. *et al.* Integrative annotation of human large intergenic noncoding RNAs reveals global properties and specific subclasses. *Genes Dev* **25**, 1915-1927, doi:10.1101/gad.17446611 (2011).

8 Kretz, M. *et al.* Suppression of progenitor differentiation requires the long noncoding RNA ANCR. *Genes Dev* **26**, 338-343, doi:gad.182121.111 [pii]10.1101/gad.182121.111 (2012).

9 Wilming, L. G. *et al.* The vertebrate genome annotation (Vega) database. *Nucleic Acids Res.* **36**, D753-760, doi:10.1093/nar/gkm987 (2008).

10 Zhang, Y., Liu, X. S., Liu, Q. R. & Wei, L. Genome-wide in silico identification and analysis of cis natural antisense transcripts (cis-NATs) in ten species. *Nucleic Acids Res* **34**, 3465-3475, doi:10.1093/nar/gkl473 (2006).

11 Romualdi, C., Bortoluzzi, S. & Danieli, G. A. Detecting differentially expressed genes in multiple tag sampling experiments: comparative evaluation of statistical tests. *Hum Mol Genet* **10**, 2133-2141 (2001).

12 Cattoglio, C. *et al.* High-definition mapping of retroviral integration sites defines the fate of allogeneic T cells after donor lymphocyte infusion. *PLoS One* **5**, e15688, doi:10.1371/journal.pone.0015688 (2010).

13 Cui, K. *et al.* Chromatin signatures in multipotent human hematopoietic stem cells indicate the fate of bivalent genes during differentiation. *Cell Stem Cell* **4**, 80-93 (2009).

14 Langmead, B., Trapnell, C., Pop, M. & Salzberg, S. L. Ultrafast and memory-efficient alignment of short DNA sequences to the human genome. *Genome Biol* **10**, R25 (2009).

15 Li, H. *et al.* The Sequence Alignment/Map format and SAMtools. *Bioinformatics (Oxford, England)* **25**, 2078-2079, doi:10.1093/bioinformatics/btp352 (2009).

16 Quinlan, A. R. & Hall, I. M. BEDTools: a flexible suite of utilities for comparing genomic features. *Bioinformatics (Oxford, England)* **26**, 841-842, doi:10.1093/bioinformatics/btq033 (2010).

17 Simon Andrews (2010). FastQC: A quality control tool for high throughput sequence data. Babraham Institute, C., UK. URL http://www.bioinformatics.babraham.ac.uk/projects/fastqc/.

18 Kharchenko, P. V., Tolstorukov, M. Y. & Park, P. J. Design and analysis of ChIP-seq experiments for DNA-binding proteins. *Nat. Biotechnology* **26**, 1351-1359 (2008).

19 Landt, S. G. *et al.* ChIP-seq guidelines and practices of the ENCODE and modENCODE consortia. *Genome Res.* **22**, 1813-1831, doi:10.1101/gr.136184.111 (2012).

20 Zang, C. *et al.* A clustering approach for identification of enriched domains from histone modification ChIP-Seq data. *Bioinformatics (Oxford, England)* **25**, 1952-1958, doi:10.1093/bioinformatics/btp340 (2009).
21 Whyte, W. A. *et al.* Master transcription factors and mediator establish super-enhancers at key cell identity genes. *Cell* **153**, 307-319, doi:10.1016/j.cell.2013.03.035 (2013).
22 Loven, J. *et al.* Selective inhibition of tumor oncogenes by disruption of super-enhancers. *Cell* **153**, 320-334, doi:10.1016/j.cell.2013.03.036 (2013).
23 Cattoglio, C. *et al.* High-definition mapping of retroviral integration sites identifies active regulatory elements in human multipotent hematopoietic progenitors. *Blood* **116**, 5507-5517, doi:10.1182/blood-2010-05-283523 (2010).
